# AKR1B10 dictates c-Myc stability to suppress colorectal cancer metastasis via PP2A nitration

**DOI:** 10.1126/sciadv.adv6937

**Published:** 2025-08-22

**Authors:** Xiaoxue Wu, Shaoqing Huang, Jialing Gao, Shuting Huang, Lulu Chen, Ziyi Zhao, Ruihan Pu, Xiaojing Ma, Xianzhi Liu, Weiling He, Mei Song

**Affiliations:** ^1^ Department of Gastrointestinal Surgery, The First Affiliated Hospital, Sun Yat-Sen University, Guangzhou, Guangdong 510275, China.; ^2^ Institute of Precision Medicine, The First Affiliated Hospital, Sun Yat-Sen University, Guangzhou, Guangdong 510275, China.; ^3^ Center of Hepato-Pancreatico-Biliary Surgery, The First Affiliated Hospital, Sun Yat-sen University, Guangzhou, Guangdong 510275, China.; ^4^ School of Public Health, Sun Yat-sen University, Guangzhou, Guangdong 510275, China.; ^5^ Department of Microbiology and Immunology, Weill Cornell Medicine, New York, NY 10065, USA.; ^6^ Department of Gastrointestinal Surgery, Xiang'an Hospital of Xiamen University, School of Medicine, Xiamen University, Xiamen, Fujian 361000, China.

## Abstract

Metabolic enzymes, critical for cellular homeostasis, are frequently co-opted in a disease-specific manner to drive cancer progression. Here, we identify aldo-keto reductase family 1 member B10 (AKR1B10), down-regulated in gastrointestinal cancers, as a pivotal metastasis suppressor correlating with improved colorectal cancer (CRC) prognosis. Mechanistically, AKR1B10 activates protein phosphatase 2A (PP2A) by preventing redox-regulated nitration of its B56α subunit, preserving holoenzyme assembly and enabling c-Myc dephosphorylation at serine-62. Loss of AKR1B10 disrupts this pathway, stabilizing c-Myc, which drives integrin signaling and metastatic dissemination in CRC. We further demonstrate that lysine-125 of AKR1B10 is essential for its interaction with PP2A-Cα and B56α nitration, thereby attenuating CRC metastatic aggressiveness. Pharmacological restoration of PP2A activity effectively mitigates metastasis associated with AKR1B10 loss. In addition, c-Myc transcriptionally represses AKR1B10, establishing a feedback loop that sustains its down-regulation and enhances metastatic progression. This study uncovers an antimetastatic mechanism involving AKR1B10-mediated PP2A activation and highlights its potential as a biomarker and therapeutic target.

## Teaser

Loss of AKR1B10 in colorectal cancer drives metastasis by disrupting PP2A-mediated c-Myc inactivation.

## INTRODUCTION

Colorectal cancer (CRC) ranks as the third most prevalent malignancy worldwide, with high rates of relapse and mortality (*1*). Despite notable advancements in CRC therapeutics, metastasis and recurrence remain major challenges to improving patient outcomes (*2*). Approximately 60 to 70% of CRC cases are diagnosed at advanced stages, with only 30 to 35% of patients surviving for more than 3 years and fewer than 20% achieving 5-year survival (*3*, *4*). The elevated abnormal gene expression, high mutational burden, extensive molecular heterogeneity, and tumor cell plasticity of CRC collectively pose tremendous obstacles in treating metastatic disease (*2*). Elucidating the molecular mechanisms driving metastasis is critical for developing biomarker-driven, personalized therapeutic strategies.

Protein phosphatase 2A (PP2A) is a ubiquitously expressed, multifaceted serine threonine phosphatase comprising a catalytic (C), scaffold (A), and a highly diverse B regulatory subunit. PP2A’s dephosphorylation activity is essential for embryogenesis and key cellular processes, including signal transduction, cell proliferation, apoptosis, and metabolic regulation (*5*–*7*). Widely recognized as a celebrated tumor suppressor, PP2A is frequently altered or inactivated in various malignancies, leading to aberrant hyperphosphorylation of oncogenic signaling molecules, such as constitutive RAS activity (*8*–*10*). This dysfunction synergizes with other oncogenic events, contributing to cancer progression, metastasis, and therapy resistance (*11*–*16*). The structural diversity of PP2A’s regulatory B subunits determines substrate specificity, enabling the phosphatase to target key tumorigenic regulators such as extracellular signal–regulated kinase (ERK), AKT, p53, Bcl-2, and c-Myc (*17*–*20*). These B subunits are classified into four distinct families: PPP2R5/B56, PPP2R2/B55, PPP2R3/PR72/PR130, and Striatin (*21*), and their accessibility to bind the core AC dimer, along with posttranslational modifications across PP2A subunits, dynamically fine-tunes PP2A’s functional versatility (*22*). For example, methylation at L309 on the C subunit facilitates selective binding of B55/B56 family members to the AC dimer (*23*–*25*). Nitration of B subunits suppresses holoenzyme assembly (*18*, *26*). Thus, PP2A dysregulation profoundly reprograms the cancer proteome, positioning PP2A as an appealing therapeutic target.

Aldo-keto reductase family 1 member B10 (AKR1B10) is a reduced form of nicotinamide adenine dinucleotide phosphate (NADPH)–dependent monomeric enzyme that belongs to the AKR 1B subfamily (*27*, *28*). It catalyzes the reduction of carbonyl substrates, including aldehydes, lipid peroxidation products, and xenobiotics, thereby protecting cells from carbonyl-induced toxicity (*29*–*31*). In addition, AKR1B10 attenuates retinoic acid signaling through retinaldehyde reduction (*32*, *33*) and recycles farnesal or geranylgeraniol, intermediates in cholesterol synthesis (*34*). While AKR1B10 is highly up-regulated in malignancies such as hepatocellular carcinoma (HCC), pancreatic carcinoma, cholangiocarcinoma, lung cancer, and breast cancer, where it promotes oncogenesis and drug resistance (*28*, *35*–*38*), it is paradoxically down-regulated in gastrointestinal cancers (*39*). Despite being recognized as a prognostic marker and therapeutic target, particularly in HCC (*40*), the implications of AKR1B10 down-regulation in gastrointestinal cancer progression remain poorly understood.

In this study, we demonstrate that AKR1B10 suppresses gastrointestinal cancer metastasis through redox-dependent posttranslational modification of the PP2A–c-Myc axis. Loss of AKR1B10 disrupts PP2A holoenzyme assembly, sustaining phosphorylation of c-Myc at serine-62 (S62), which promotes epithelial-mesenchymal transition (EMT) and enhances the metastatic potential of CRC. Furthermore, activated c-Myc transcriptionally represses AKR1B10, creating a self-sustaining feedback loop. These findings reveal AKR1B10 as a critical regulator of PP2A enzymatic activity and underscore the therapeutic potential of restoring PP2A activity to counteract AKR1B10 loss–driven CRC metastasis.

## RESULTS

### Down-regulation of AKR1B10 correlates with CRC progression and poor prognosis

**Fig. 1.**
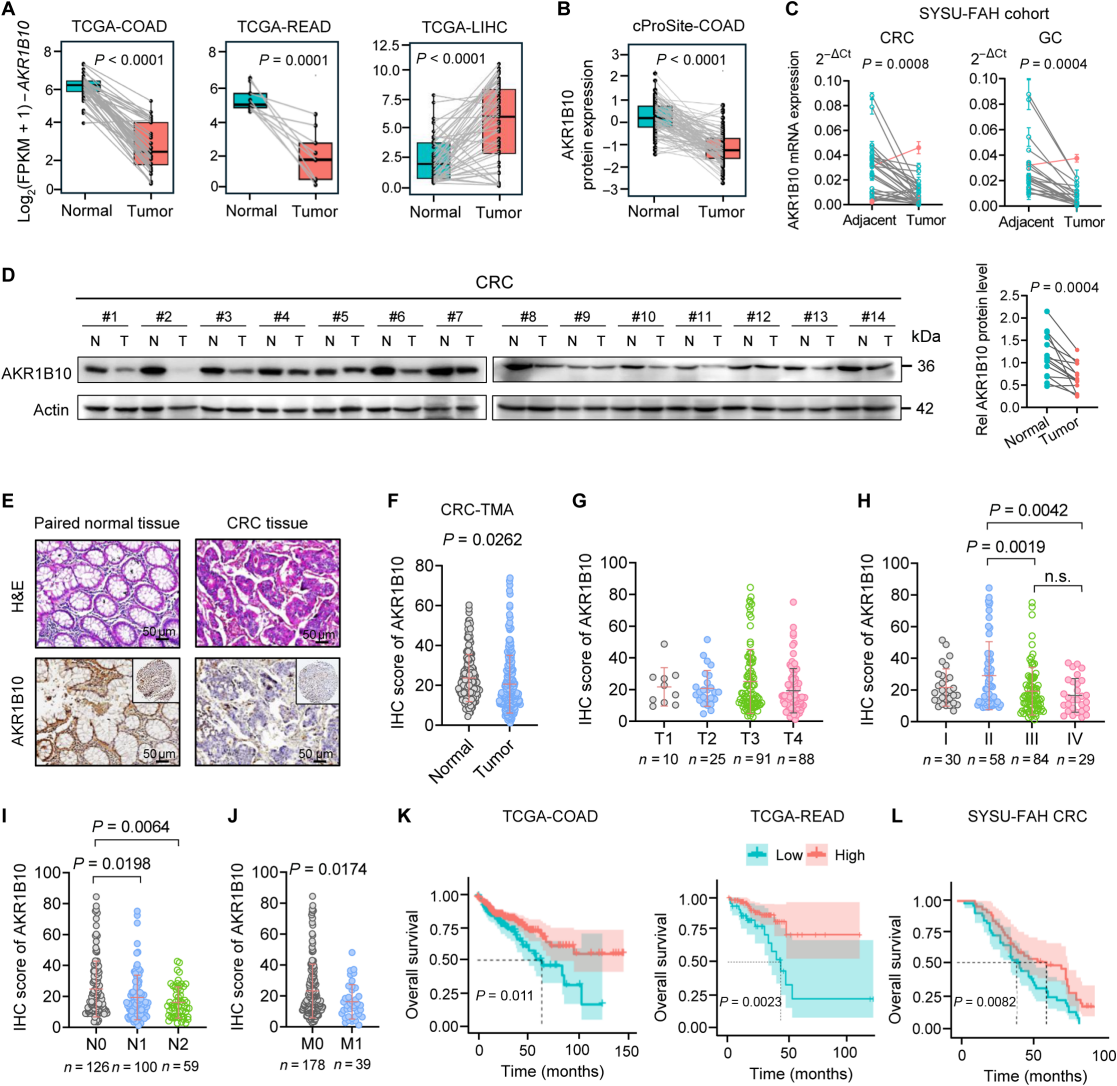
AKR1B10 down-regulated in CRC correlates with poor prognosis. (**A**) Paired analysis of *AKR1B10* mRNA expression in adjacent normal tissues versus primary tumor samples from TCGA colon adenocarcinoma (COAD; *n* = 41), rectum adenocarcinoma (READ; *n* = 9), and liver HCC (LIHC; *n* = 49) database. FPKM, fragments per kilobase of exon model per million mapped fragments. (**B**) Protein expression of AKR1B10 in COAD tissues compared to paired normal tissues based on cProSite database (*n* = 96). (**C**) Quantitative polymerase chain reaction (qPCR) analysis of *AKR1B10* mRNA expression in paired CRC (*n* = 30)/GC (*n* = 21) tissues and adjacent normal tissues from SYSU-FAH. (**D**) Western blot analysis of AKR1B10 protein expression in 14 paired adjacent normal tissues (N) and CRC tissues (T) from SYSU-FAH. Rel, relative. (**E** and **F**) Representative IHC staining (E) and quantification (F) of AKR1B10 expression in CRC tumor microarrays (TMAs). Scale bars, 50 μm. (**G** to **J**) Analysis of AKR1B10 protein expression in CRC TMAs stratified by T stages (G), National Comprehensive Cancer Network stages (H), lymph node metastases (I), and distant metastases (J). n.s., not significant. (**K** and **L**) Kaplan-Meier overall survival curves of patients with low versus high AKR1B10 expression, derived from TCGA-COAD (*n* = 430)/READ (*n* = 154) databases (K), and IHC analysis of patients with CRC from SYSU-FAH (L) (*n* = 93). Data are analyzed with unpaired Student’s *t* test (F and H to J), paired Student’s *t* test (A to D), and log-rank test (K and L).

To assess the clinical relevance of AKR1B10 in CRC, we analyzed its mRNA and protein expression levels through The Cancer Genome Atlas (TCGA) and cProSite databases. Unlike its up-regulation in HCC, breast cancer, and lung cancer, AKR1B10 was down-regulated in CRC and gastric cancer (GC) tissues (Fig. 1, A and B, and fig. S1, A and B). This finding was validated in paired CRC and GC tissue samples from the First Affiliated Hospital of Sun Yat-sen University (SYSU-FAH) (Fig. 1, C and D, and fig. S1C) and further corroborated by immunohistochemical (IHC) analysis of tissue microarrays (TMAs) comprising 217 paired CRC tissue samples (Fig. 1, E and F, and table S1). Reduced AKR1B10 expression was strongly associated with advanced stages and higher incidence of lymph nodes and distant organ metastases (Fig. 1, G to J, and fig. S1, D and E). Survival analyses from TCGA, Gene Expression Omnibus (GEO), and SYSU-FAH datasets consistently demonstrated that low AKR1B10 expression predicted poor outcomes in patients with CRC and GC (Fig. 1, K and L, and fig. S1, F to H). Furthermore, multivariate analysis identified AKR1B10 as an independent prognostic factor for CRC (fig. S1I). These data establish a clear inverse relationship between AKR1B10 expression and CRC aggressiveness and prognosis.

### *AKR1B10* deficiency promotes CRC metastasis

**Fig. 2.**
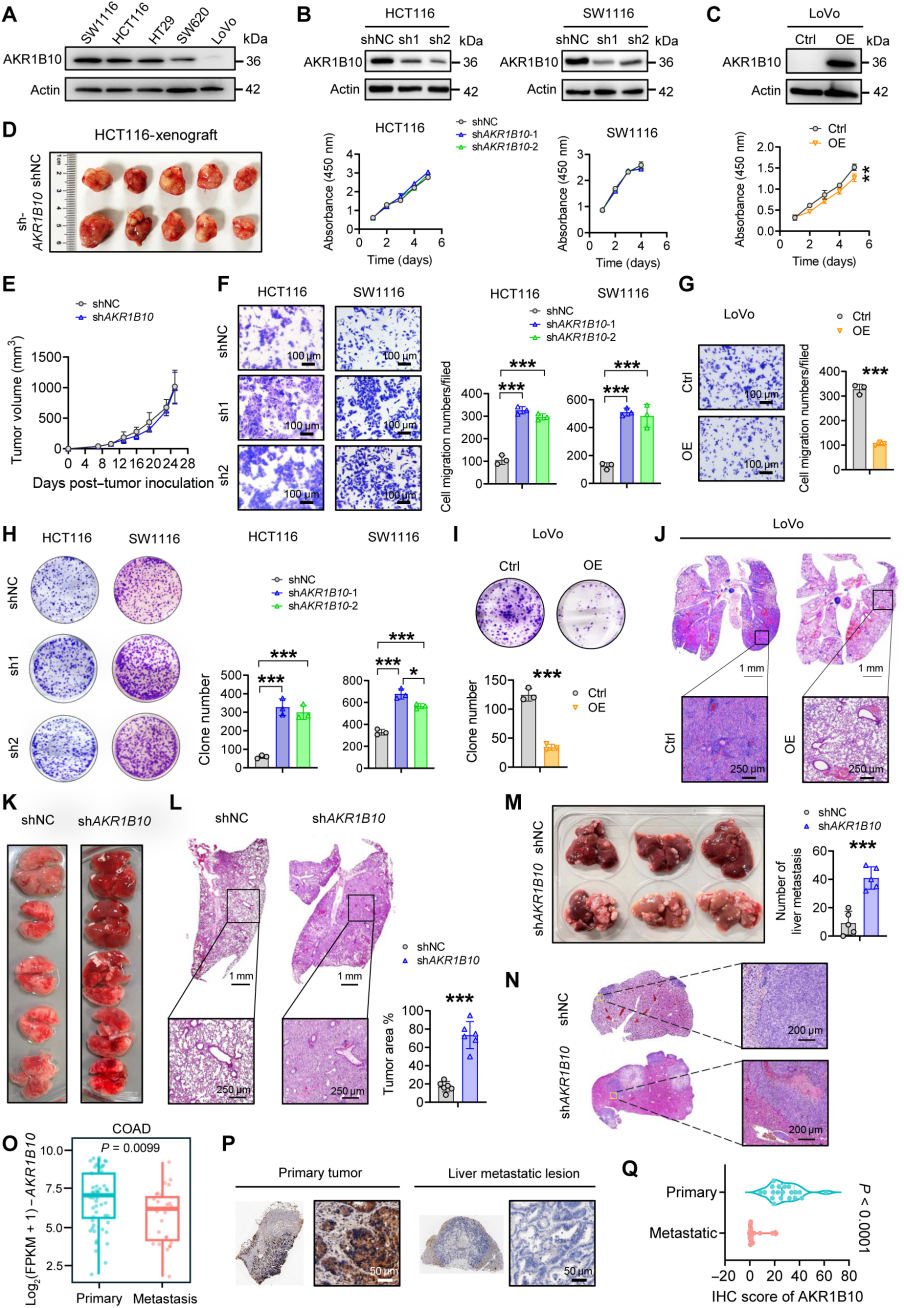
AKR1B10 curbs CRC metastasis in vitro and in vivo. (**A**) Western blot analysis of AKR1B10 protein levels in human CRC (hCRC) cell lines. (**B**) Validation of AKR1B10 knockdown efficiency in HCT116 and SW1116 cells via Western blot and assessment of cell proliferation with Cell Counting Kit-8 (CCK-8) assays (*n* = 4). (**C**) Verification of AKR1B10 overexpression in LoVo cells by Western blot, with corresponding CCK-8 proliferation analysis (*n* = 4). (**D** and **E**) Representative images (D) and statistical analysis (E) of xenograft tumor volumes in nude mice implanted with HCT116 (shNC&sh*AKR1B10*) cells (*n* = 5 mice per group). (**F** and **G**) Representative transwell assay images (left) and quantification (right) in HCT116/SW1116 cells (F) or LoVo cells after 48 hours (G). (**H** and **I**) Representative clonogenic assay images and quantification in HCT116/SW1116 cells (H) or LoVo cells (I). (**J**) H&E staining of lung metastatic nodules following intravenous injection of LoVo (Ctrl&AKR1B10-OE) cells. Ctrl, control; OE, overexpression. Scale bars, 250 μm. (**K** and **L**) Representative images (K) and quantification of lung tumor nodule area per section (L) in nude mice intravenously injected with HCT116 (shNC&sh*AKR1B10*) cells (*n* = 5 mice per group). (**M** and **N**) Representative images and quantification of liver metastatic nodules in nude mice after splenic injection of HCT116 (shNC&sh*AKR1B10*) cells (M), with corresponding H&E staining of liver metastatic lesions (N) (*n* = 5 mice per group). (**O**) mRNA expression levels of AKR1B10 in primary tumors (*n* = 56) and metastases (*n* = 27; liver = 23, peritoneum = 3, and lung = 1) based on GSE28702. (**P** and **Q**) Representative IHC images (P) and quantification (Q) of AKR1B10 in paired primary CRC tissues and liver metastatic lesions from 20 patients. Scale bars, 50 μm. (F to I) *n* = 3. Data are presented as mean ± SD, with two-way analysis of variance (ANOVA) test in (C) and (E) or unpaired Student’s *t* test (F to I, L, M, O, and Q). *P < 0.05, **P < 0.01, and ***P < 0.001.

To explore the functional role of AKR1B10 in CRC progression, we ectopically overexpressed or silenced *AKR1B10* in CRC cell lines, including SW1116, HCT116, and LoVo (Fig. 2A). Of them, SW1116 is microsatellite stable, HCT116 is microsatellite instability high (MSI-H), and LoVo displays a unique hybrid profile with MSI-low features. All three cell lines constitutively express the oncoprotein c-Myc. Knockdown of *AKR1B10* marginally affected CRC cell propagation both in vitro and in vivo, whereas overexpression of AKR1B10 slightly enhanced CRC cell proliferation (Fig. 2, B to E, and fig. S2A). Moreover, AKR1B10 depletion did not alter cell cycle progression but led to a modest reduction in apoptosis in CRC cells (fig. S2, B to E). AKR1B10 deficiency markedly increased cell migration, colony formation, and wound healing abilities compared to control groups (Fig. 2, F and H, and fig. S2, F and G). Conversely, AKR1B10 overexpression suppressed these aggressive traits (Fig. 2, G and I, and fig. S2H). We also observed similar results in GC cell lines (MKN45 and AGS) (fig. S2, I to O). To confirm these observations in vivo, we used an intravenous injection model of pulmonary metastasis and an intrasplenic injection model for liver metastasis. Mice injected with AKR1B10-overexpressing LoVo cells exhibited fewer lung metastatic foci (Fig. 2J), while those receiving AKR1B10-deficient HCT116 cells showed a marked increase in metastatic nodules in both lung and liver tissues, as evidenced by ex vivo quantification and hematoxylin and eosin (H&E) staining (Fig. 2, K to N). The AKR1B10 expression was markedly down-regulated in metastatic lesions compared to primary tumors, as evidenced by analyses of the colon adenocarcinoma (COAD)–GEO dataset and our independent cohort (Fig. 2, O to Q). These findings collectively demonstrate that AKR1B10 acts as a suppressor of CRC metastasis irrespective of microsatellite instability status.

### AKR1B10 curbs CRC metastasis by repressing integrin transcription

**Fig. 3.**
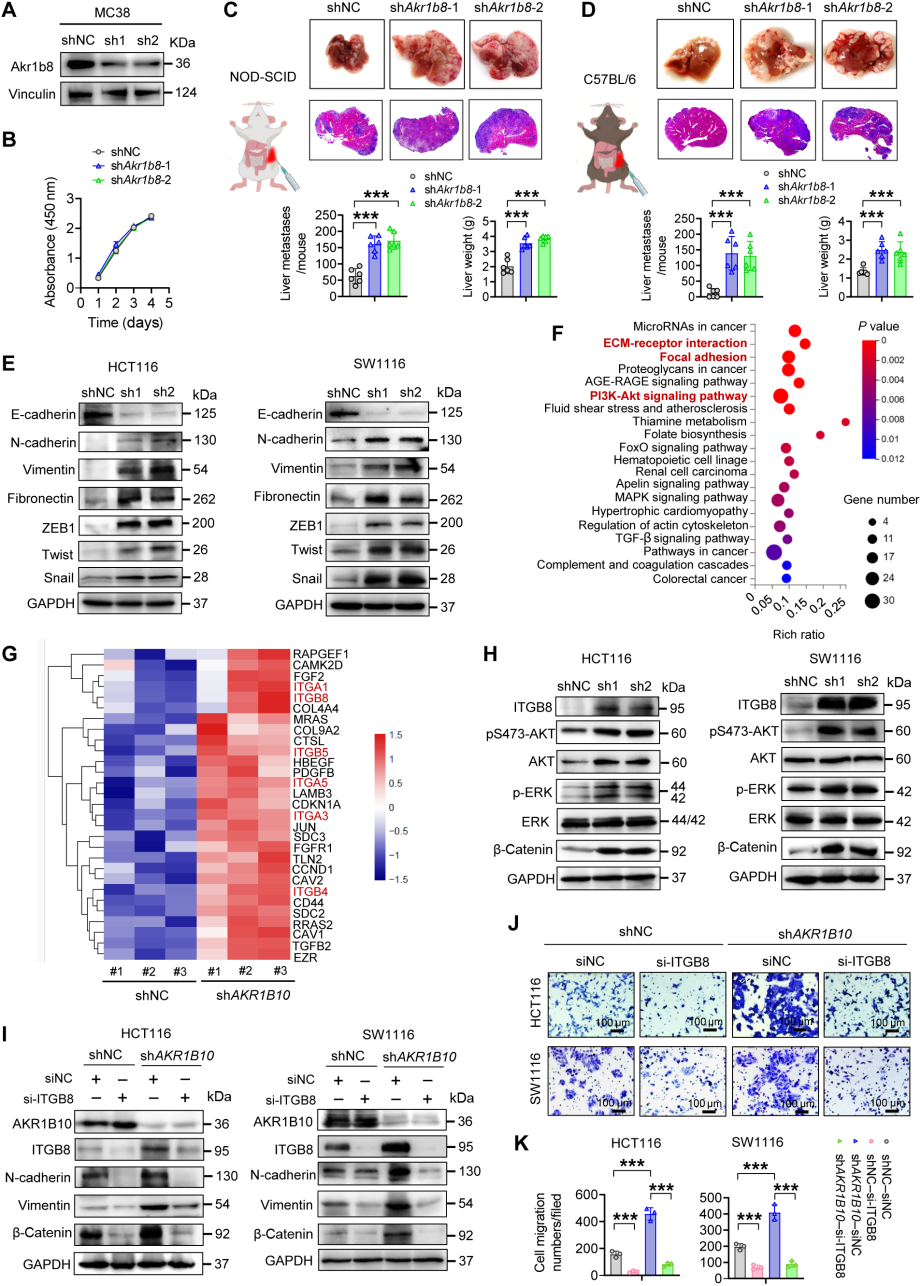
AKR1B10 suppresses EMT by down-regulating integrin signaling. (**A**) Western blot analysis of Akr1b8 knockdown efficiency in MC38 cells. (**B**) Cell proliferation rates of MC38 (shNC and sh*Akr1b8*) cells assessed using the CCK-8 assay (*n* = 4). (**C**) Representative images of liver metastatic nodules in NOD-SCID mice on day 15 following splenic injection of MC38 shNC and sh*Akr1b8* cells. Liver weight and the number of metastases were quantified (*n* = 6 mice per group). (**D**) Representative images of liver metastatic nodules in C57BL/6 mice on day 18 following splenic injection of MC38 (shNC and sh*Akr1b8*) cells. Liver weight and the number of metastases were quantified (*n* = 6 mice per group). (**E**) Western blot analysis of EMT markers and EMT-related TFs in HCT116 and SW1116 cells. GAPDH, glyceraldehyde-3-phosphate dehydrogenase. (**F**) Kyoto Encyclopedia of Genes and Genomes (KEGG) pathway enrichment analysis of the top 20 up-regulated biological processes in sh*AKR1B10* cells based on RNA sequencing (RNA-seq). PI3K, phosphatidylinositol 3-kinase; MAPK, mitogen-activated protein kinase; TGF-β, transforming growth factor–β; AGE-RAGE, Advanced Glycation End products–Receptor for Advanced Glycation End products; FoxO, Forkhead box O. (**G**) Heatmap depicting mRNA levels of genes from selected KEGG pathways. (**H**) Western blot analysis of ITGB8 protein and its downstream factors in HCT116 and SW1116 cells. p-ERK, phosphorylated ERK. (**I**) Western blot analysis of ITGB8 protein and its downstream EMT markers in HCT116 and SW1116 cells after ITGB8 silencing. (**J** and **K**) Representative images (J) and quantification (K) of transwell assays in HCT116 and SW1116 cells after ITGB8 silencing. (C, D, and K) *n* = 3. Data are presented as mean ± SD, with two-way ANOVA test (B) or unpaired Student’s *t* test (C, D, and K). ***P < 0.001.

While AKR1B10 has been implicated in reducing glycolytic capacity in breast cancer cells, supporting their survival under low glucose conditions and facilitating metastatic colonization in the lung (*41*), our Seahorse XF Glycolysis Stress tests showed that AKR1B10 depletion did not modulate compensatory glycolysis but attenuated basal glycolytic activity in CRC/GC cell lines, suggesting cancer type–specific metabolic adaptation mechanisms (fig. S3A). Similarly, down-regulation of Akr1b8, the murine ortholog of human *AKR1B10*, did not affect MC38 cell proliferation in vitro (Fig. 3, A and B). Notably, tumors derived from MC38-sh*Akr1b8* cells exhibited a notable increase in liver metastasis in both nonobese diabetic (NOD)–severe combined immunodeficient (SCID) and C57BL/6 mouse models (Fig. 3, C and D), suggesting that AKR1B10 and Akr1b8 suppress CRC metastasis via cell-intrinsic mechanisms.

The EMT is a crucial process conducive to tumor dissemination (*42*). Upon AKR1B10 depletion, CRC cells underwent a morphological shift from cuboidal epithelial shapes to elongated mesenchymal forms (fig. S3B), accompanied by reduced E-cadherin levels and increased expression of mesenchymal markers, including N-cadherin, vimentin, and fibronectin, as well as EMT drivers such as Twist, ZEB1, and Snail (Fig. 3E). RNA sequencing (RNA-seq) transcriptome profiling further revealed up-regulation of cell adhesion–related pathways, including extracellular matrix (ECM)–receptor interactions and focal adhesion, in sh*AKR1B10* cells (Fig. 3, F and G). Among these pathways, integrins emerged as prominently up-regulated, with TCGA database analysis corroborating an inverse correlation between AKR1B10 expression and integrin gene expression in CRC and GC tissues (fig. S3C). Downstream integrin pathways, such as AKT and ERK signaling, were activated in AKR1B10-deficient cells, along with a notable up-regulation of ITGB8 expression (Fig. 3H and fig. S3D). Knocking down ITGB8 in CRC cells reduced mesenchymal marker expression (e.g., N-cadherin and vimentin) at both mRNA and protein levels, effectively reversing the migratory and colonization advantages conferred by AKR1B10 abrogation (Fig. 3, I to K, and fig. S3, E and F). Similar results were observed in GC cells (fig. S4). Together, these findings suggest that AKR1B10 loss enhances CRC cell migratory and invasive potential through integrin transcriptional induction.

### AKR1B10 transcriptionally regulates integrins by modulating c-Myc stability

**Fig. 4.**
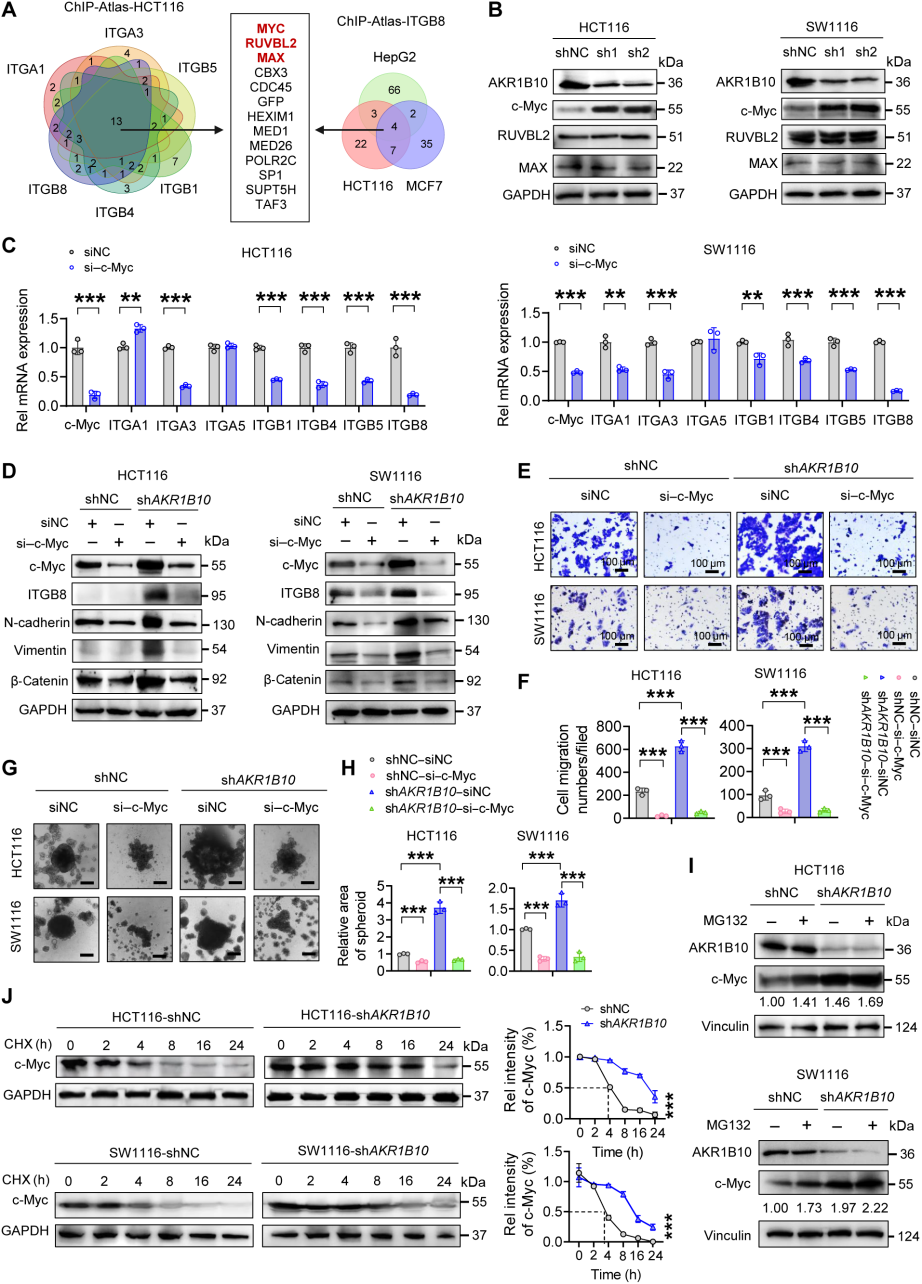
AKR1B10 destabilizes c-Myc to repress integrin transcription. (**A**) The Venn diagram showing three TFs capable of binding to integrin gene promoter regions in HCT116 cells and to the *ITGB8* promoter region in various cancers, based on the ChIP-Atlas database. (**B**) Western blot analysis of c-Myc, RUVBL2, and MAX protein expression in HCT116 and SW1116 (shNC&sh*AKR1B10*) cells. (**C**) qPCR analysis of integrin mRNA expression in HCT116 and SW1116 cells after c-Myc silencing. (**D**) Western blot analysis of ITGB8 protein and its downstream EMT markers in HCT116 and SW1116 cells after c-Myc silencing. (**E** and **F**) Representative images (E) and quantification (F) of transwell assay in HCT116 and SW1116 cells after c-Myc silencing. (**G** and **H**) Representative images (G) and quantification (H) of spheroid formation assays in HCT116 and SW1116 cells after c-Myc silencing. Scale bars, 50 μm. (**I**) Western blot analysis of c-Myc expression in HCT116 and SW1116 (shNC&sh*AKR1B10*) cells after treatment with MG132 (10 μM for 8 hours). c-Myc protein levels were quantified and normalized to loading control (vinculin). (**J**) Detection of c-Myc protein turnover in HCT116 and SW1116 cells by Western blot after CHX (100 μg/ml) treatment and quantified by ImageJ software. h, hours. (C, F, H, and J) *n* = 3. Data are presented as mean ± SD, with unpaired Student’s *t* test (C, F, and H) or with two-way ANOVA test (J). **P < 0.01 and ***P < 0.001.

To identify transcription factors (TFs) mediating AKR1B10’s regulation of integrin expression, we analyzed proteins that bind integrin gene promoters in HCT116 cells and the *ITGB8* promoter across various tumor cell lines using the chromatin immunoprecipitation (ChIP)–Atlas database. This analysis identified three candidates: c-Myc, RUVBL2, and MAX (Fig. 4A). Notably, AKR1B10 depletion increased c-Myc protein levels in CRC and GC cells, while RUVBL2 and MAX levels remained unchanged (Fig. 4B and fig. S5, A and B). Furthermore, c-Myc knockdown substantially reduced mRNA levels of integrins, particularly *ITGB8*, while silencing RUVBL2 had minimal effects. Knocking down MAX, an obligate dimerization partner of c-Myc, displayed a similar suppressive effect on integrin expression, supporting the role of the c-Myc–MAX heterodimer as the transcriptional driver of integrin genes in gastrointestinal tumor cells (Fig. 4C and fig. S5, C to E). Depleting c-Myc in sh*AKR1B10* CRC/GC cells also suppressed mesenchymal marker expression and impaired their migratory capacity and anchorage-independent and anchorage-dependent growth (Fig. 4, D to H, and fig. S5, F to I). c-Myc has a short half-life that is rigorously controlled by phosphorylation and proteasomal degradation (*43*). Treatment with the proteasome inhibitor MG132 elevated c-Myc levels in control cells but had less effect in AKR1B10-deficient cells, suggesting that AKR1B10 loss hinders the proteasomal degradation of c-Myc (Fig. 4I and fig. S5J). Cycloheximide (CHX) chase assays further revealed a reduced c-Myc protein decay rate in AKR1B10-deficient cells, supporting its role in c-Myc stabilization (Fig. 4J and fig. S5K). Together, these data demonstrate that AKR1B10 facilitates c-Myc proteasomal degradation, thereby repressing integrin transcription and suppressing metastatic traits in CRC.

### AKR1B10 destabilizes c-Myc by facilitating PP2A holoenzyme assembly

**Fig. 5.**
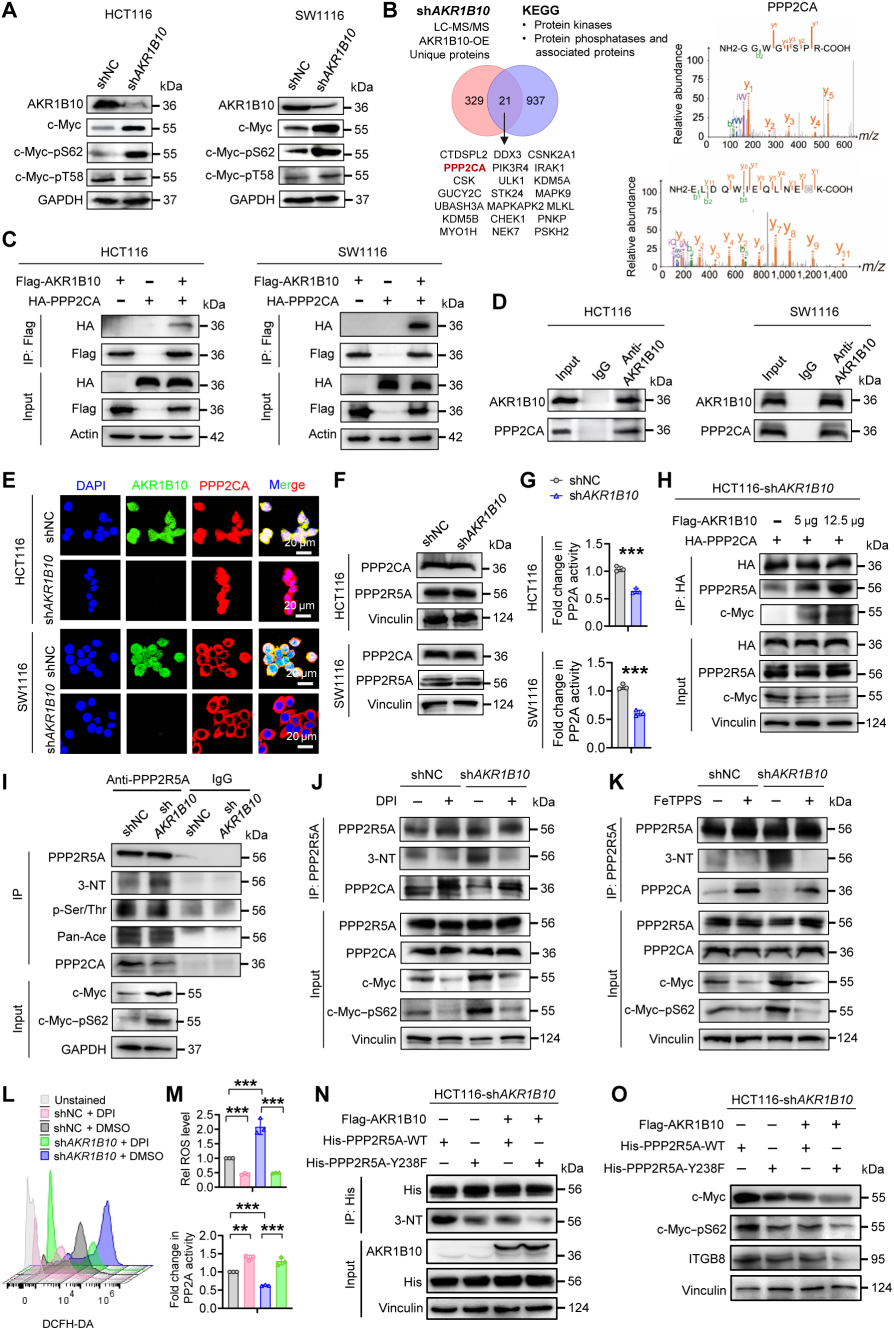
Targeting AKR1B10 enhances c-Myc stability by disrupting PP2A assembly. (**A**) Western blot analysis of c-Myc protein expression, and its phosphorylation status in HCT116 and SW1116 (shNC&sh*AKR1B10*) cells. (**B**) IP-MS analysis to identify AKR1B10-interacting kinases and phosphatases. *m/z*, mass/charge ratio. (**C**) Co-IP analysis showing the physical interaction of exogenous AKR1B10 with PPP2CA in HCT116 and SW1116 cells. (**D**) Physical interaction of endogenous AKR1B10 with PPP2CA was detected by Co-IP with anti-AKR1B10 antibody in HCT116 and SW1116 cells. (**E**) Immunofluorescent staining showing the colocalization of endogenous AKR1B10 with PPP2CA in HCT116 and SW1116 cells. Scale bars, 20 μm. DAPI, 4′,6-diamidino-2-phenylindole. (**F**) Western blot analysis of PPP2CA and PPP2R5A protein expression in HCT116 and SW1116 (shNC&sh*AKR1B10*) cells. (**G**) PP2A activity assay in HCT116 and SW1116 (shNC&sh*AKR1B10*) cells (*n* = 3). (**H**) Co-IP analysis of HA-PPP2CA in HCT116 cells coexpressing Flag-AKR1B10 with varying plasmid amounts, as indicated. (**I**) Co-IP analysis of PPP2R5A indicating increased 3-nitrotyrosine (3-NT) levels and reduced PPP2CA interaction in HCT116-sh*AKR1B10* cells. Pan-Ace, Pan Acetylation. (**J** and **K**) Co-IP analysis of PPP2R5A showing reduced 3-NT levels and increased interaction with PPP2CA in the presence of DPI [10 μM for 20 hours (J)] or FeTPPS [10 μM for 3 hours (K)] in HCT116 (shNC&sh*AKR1B10*) cells. (**L** and **M**) Representative fluorescence-activated cell sorting (FACS) images (L) and quantification of intracellular reactive oxygen species (ROS) levels, and relative PP2A activities [(M); *n* = 3] in HCT116 (shNC&sh*AKR1B10*) cells treated with DPI (10 μM for 20 hours). DMSO, dimethyl sulfoxide. (**N** and **O**) His-PPP2R5A-WT or His-PPP2R5A-Y238F plasmid was transfected into HCT116-sh*AKR1B10* cells with or without Flag-AKR1B10. Co-IP analysis showing reduced 3-NT levels of His-PPP2R5A-Y238F (N). Western blot demonstrating decreased total c-Myc, pS62 c-Myc, and reduced ITGB8 expression in the presence of His-PPP2R5A-Y238F (O). Data in (G) and (M) are presented as mean ± SD, with unpaired Student’s *t* test. **P < 0.01 and ***P < 0.001.

The stability of c-Myc is meticulously regulated by phosphorylation at S62 and threonine-58 (T58). Phosphorylation at S62 (pS62) stabilizes c-Myc, while its dephosphorylation by PP2A enables proteasomal degradation of phospho-T58 (pT58) Myc (*44*, *45*). In AKR1B10-depleted CRC/GC cells, we observed elevated levels of pS62 c-Myc and total c-Myc, while pT58 c-Myc remained unchanged (Fig. 5A and fig. S6A). To explore the mechanisms, immunoprecipitation–mass spectrometry (IP-MS) identified the catalytic subunit of PP2A (PPP2CA) as a top AKR1B10 interactor, consistent with Kyoto Encyclopedia of Genes and Genomes (KEGG) analysis linking AKR1B10 to posttranslational modification and signal transduction pathways (Fig. 5B and fig. S6B). Coimmunoprecipitation (Co-IP) confirmed interactions between Flag-tagged AKR1B10 and hemagglutinin (HA)–tagged PPP2CA, as well as endogenous AKR1B10 and PPP2CA in CRC/GC cells, with colocalization observed primarily in the cytosol (Fig. 5, C to E, and fig. S6, C and D). AKR1B10 loss did not affect the protein expression or proteasomal degradation of PPP2CA and B56α (PPP2R5A), the regulatory subunit known to mediate PP2A-driven c-Myc dephosphorylation (Fig. 5F and fig. S6, E to G) (*19*, *26*). However, AKR1B10 depletion substantially reduced PP2A activity (Fig. 5G and fig. S6H). Overexpression of AKR1B10 in sh*AKR1B10* CRC cells restored PPP2CA–B56α–c-Myc interactions and promoted c-Myc degradation in a dose-dependent manner (Fig. 5H and fig. S6I).

The nitration of B56α by peroxynitrite (ONOO^−^), a product of superoxide (O2^●–^) and nitric oxide (NO), inhibits PP2A holoenzyme assembly required for c-Myc dephosphorylation (*26*). Immunoprecipitated B56α lysates from CRC cells showed increased 3-nitrotyrosine (3-NT) levels and reduced B56α-PPP2CA interactions in AKR1B10-depleted cells. This was accompanied by elevated pS62 c-Myc and total c-Myc, with no changes in B56α phosphorylation (serine/threonine) or acetylation (Fig. 5I). These findings indicate that AKR1B10 deficiency fosters a prooxidant microenvironment promoting nitrative inhibition of B56α, impairing PP2A activity and c-Myc destabilization. Treatment with the NADPH oxidase inhibitor diphenyleneiodonium chloride (DPI) reduced O2^●–^ levels, mitigating B56α nitration and restoring B56α-PPP2CA interaction. DPI treatment accelerated c-Myc dephosphorylation and degradation in AKR1B10-depleted cells (Fig. 5J and fig. S6J). Similarly, Fluorescent-tagged Thiol-Protected Proteolytic Substrate (FeTPPS), a ONOO^−^ scavenger, reversed nitrative inhibition of B56α and restored PP2A activity by reducing NO production (Fig. 5K and fig. S6M). DPI and FeTPPS treatment profoundly enhanced PP2A activity, with DPI reducing intracellular reactive oxygen species (ROS) levels, countering the effects of AKR1B10 loss (Fig. 5, L and M, and fig. S6, K, L, and N to Q). Critically, the nonnitratable B56α Y238F mutant (*26*) effectively reversed AKR1B10 loss–induced B56α nitration. This mutant synergized with AKR1B10 reconstitution, amplifying the reduction in 3-NT levels (Fig. 5N and fig. S6R). Unlike wild-type (WT) B56α, the Y238F mutant failed to stabilize phosphorylated c-Myc or activate c-Myc signaling (Fig. 5O and fig. S6S). Collectively, these results demonstrate that AKR1B10 facilitates PP2A holoenzyme assembly, destabilizing c-Myc by promoting its dephosphorylation at S62.

### L125 of AKR1B10 is essential for PP2A assembly and CRC metastasis suppression

**Fig. 6.**
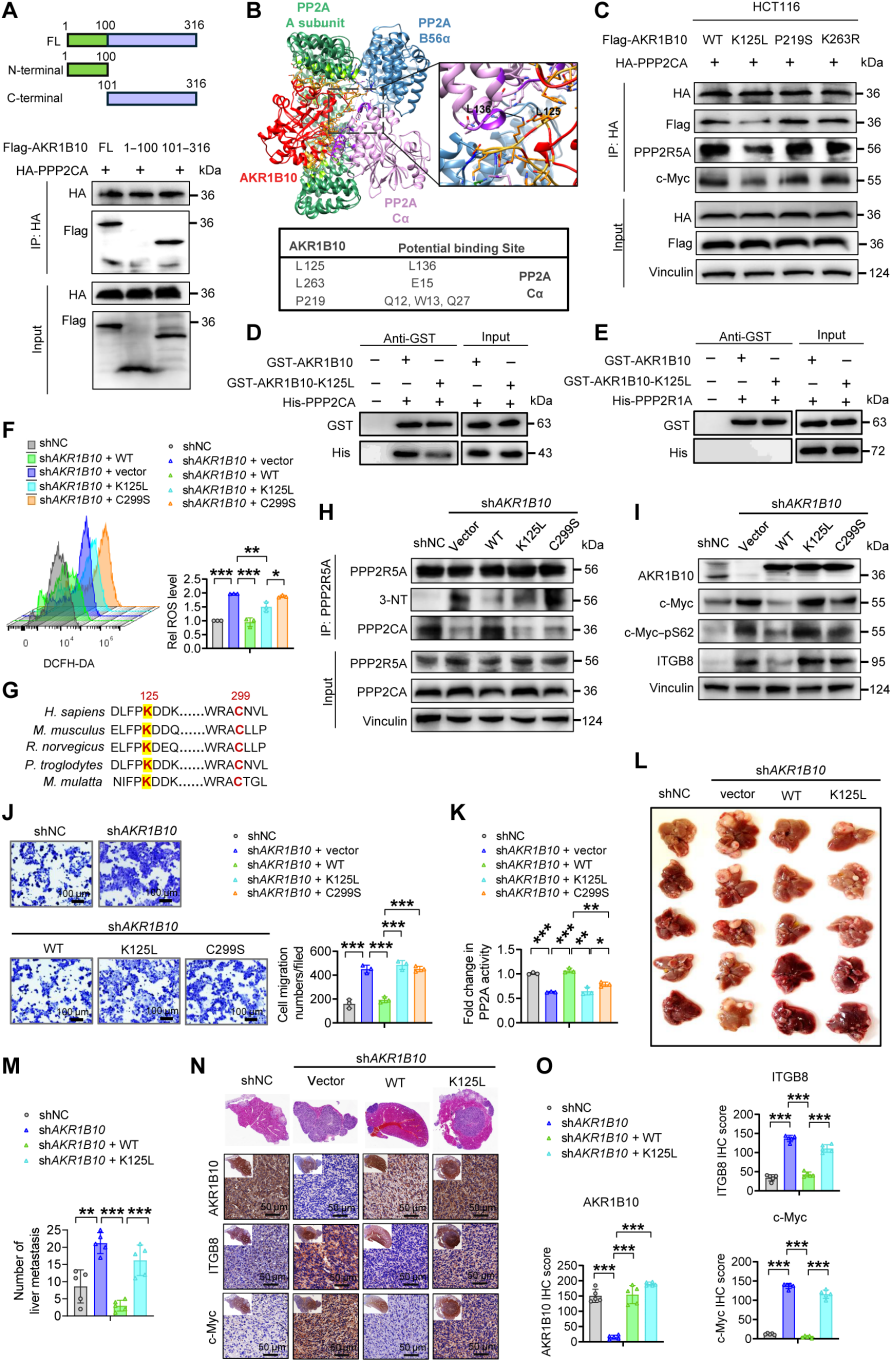
AKR1B10^K125L^ impedes PP2A assembly to drive CRC metastasis. (**A**) Schematic representation of AKR1B10 truncations and Co-IP analysis of Flag-tagged AKR1B10 truncations in HCT116 cells, as indicated. FL, full length. (**B**) Computational docking model predicting the interaction between AKR1B10 (red) and PP2A (purple) using the Global RAnge Molecular Matching (GRAMM) algorithm. (**C**) Co-IP analysis showing the interaction between PPP2CA with AKR1B10^WT^, AKR1B10^K125L^, AKR1B10^P219S^, and AKR1B10^K263R^ in HCT116 cells, using an anti-HA antibody. (**D** and **E**) Recombinant GST-tagged AKR1B10 (WT or K125L) was incubated with His-PPP2CA (D) or His-PPP2R1A (E). Bound proteins were analyzed by Western blot. Data are representative of three independent experiments. (**F**) Representative FACS images and quantification of intracellular ROS levels in HCT116 shNC and sh*AKR1B10* cells expressing mock, AKR1B10^WT^, AKR1B10^K125L^, and AKR1B10^C299S^. (**G**) Alignment of K125 and C299 amino acid residues in AKR1B10 across multiple species. *H. sapiens*, *Homo sapiens*; *M. musculus*, *Mus musculus*; *R. norvegicus*, Rattus norvegicus; *P. troglodytes*, *Pan troglodytes*; *M. mulatta*, *Macaca mulatta*. (**H**) Co-IP analysis of PPP2R5A demonstrating increased 3-NT and reduced PPP2CA interaction in AKR1B10^K125L^ and AKR1B10^C299S^ overexpressing HCT116-sh*AKR1B10* cells. (**I**) Western blot analysis of c-Myc, phospho-Myc (p-Myc), and ITGB8 protein expression in HCT116 shNC and sh*AKR1B10* cells expressing mock, AKR1B10^WT^, AKR1B10^K125L^, and AKR1B10^C299S^. (**J** and **K**) Representative images (left) and quantification (right) of transwell assay (J), and relative PP2A activity (K) in HCT116 shNC and sh*AKR1B10* cells expressing mock, AKR1B10^WT^, AKR1B10^K125L^, and AKR1B10^C299S^. (**L** to **O**) Representative images (L) and quantification (M) of liver metastatic nodules in nude mice following splenic injection of HCT116 shNC and sh*AKR1B10* cells expressing mock, AKR1B10^WT^, and AKR1B10^K125L^. Representative H&E staining and IHC images of AKR1B10, ITGB8, and c-Myc staining in liver sections (N), with quantified histoscores (O) (*n* = 5 mice per group). (F, J, and K) *n* = 3. Data in (F), (J), (K), (M), and (O) are presented as mean ± SD, with unpaired Student’s *t* test. *P < 0.05, **P < 0.01, and ***P < 0.001.

While DPI and FeTPPS partially restored B56α-PPP2CA interactions in sh*AKR1B10* CRC cells, the levels remained below those of control cells (Fig. 5, J and K). This indicates that AKR1B10 contributes to PP2A holoenzyme assembly not solely by reducing nitrative inhibition but also through direct interaction with PPP2CA. AKR1B10 was found to interact with PPP2CA independent of its N-terminal residues (1 to 100) (Fig. 6A). Protein-protein docking simulation identified potential interaction sites between AKR1B10 and the PP2A A/Cα core enzyme, rather than B56α. Specifically, there are three residues in AKR1B10 that may form interactions with corresponding amino acids in PPP2CA (Fig. 6B and fig. S7A). Among them, AKR1B10^K125L^ markedly weakened the interaction with PPP2CA, reducing its association with B56α and c-Myc, supporting our hypothesis that AKR1B10 facilitates c-Myc–bound PP2A holoenzyme assembly through direct PPP2CA binding (Fig. 6C). Furthermore, glutathione *S*-transferase (GST) pull-down assays with recombinant proteins confirmed that AKR1B10 selectively interacts with PPP2CA via the K125 residue while showing no direct binding to the PP2A Aα (PPP2R1A) (Fig. 6, D and E, and fig. S7, B and C).

AKR1B10 exhibits superior catalytic efficiency for retinaldehyde and detoxifies lipid-derived reactive carbonyls (*46*, *47*). To assess the importance of AKR1B10’s catalytic activity in PP2A assembly, we reconstituted sh*AKR1B10* CRC cells with AKR1B10 WT or catalytically impaired mutants: K125L, V301L (reducing catalytic activity for all-trans-retinaldehyde) (*32*), and C299S (targeting catalytic activity for 4-hydroxynonenal) (*47*). While AKR1B10^WT^ and AKR1B10^V301L^ normalized intracellular ROS levels, AKR1B10^K125L^ and AKR1B10^C299S^ failed to reduce ROS. K125 and C299, unlike V301, are evolutionarily conserved, underscoring their critical roles in AKR1B10’s reductase function (Fig. 6, F and G, and fig. S7, D to F). AKR1B10^WT^ reconstitution also restored B56α-PPP2CA interactions, which were impaired in cells expressing AKR1B10^K125L^ and AKR1B10^C299S^ (Fig. 6H and fig. S7G). Functionally, AKR1B10^WT^ reconstitution suppressed c-Myc and ITGB8 expressions, reduced sh*AKR1B10* cells’ migratory capability, and restored PP2A activity, eliminating off-target effects of short hairpin RNA (shRNA). However, AKR1B10^K125L^ and AKR1B10^C299S^ mutants did not achieve comparable outcomes (Fig. 6, I to K, and fig. S7, H to L). Notably, AKR1B10^K125L^ showed a stronger inhibitory effect on PP2A assembly and downstream signaling than AKR1B10^C299S^, which did not disrupt PPP2CA binding, highlighting the dual requirement of AKR1B10’s catalytic activity and direct interaction with PPP2CA for PP2A functionality (fig. S7M). In vivo, AKR1B10^WT^, but not AKR1B10^K125L^, attenuated liver metastasis of AKR1B10-deficient CRC cells in the intrasplenic injection mouse model (Fig. 6, L and M), nor did AKR1B10^K125L^ reduce c-Myc and ITGB8 expression within liver metastatic lesions (Fig. 6, N and O). These findings demonstrate that K125 is indispensable for AKR1B10-mediated PP2A assembly and metastasis suppression in CRC.

### Pharmacological activation of PP2A suppresses AKR1B10 depletion-driven metastasis

**Fig. 7.**
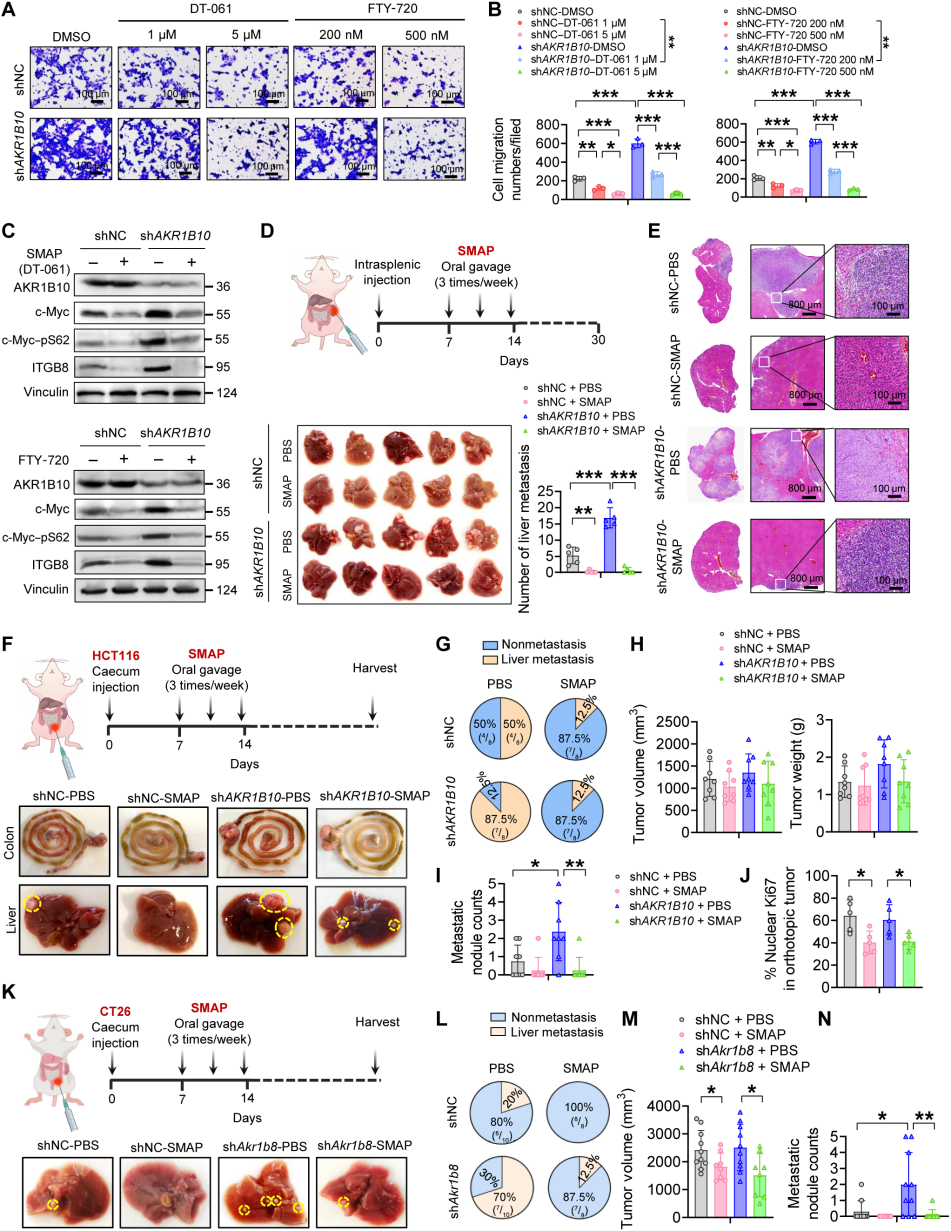
PP2A activators counteract AKR1B10 loss–driven metastasis. (**A** and **B**) Representative images (A) and quantification (B) of transwell assay in HCT116 cells treated with DT-061 or FTY-720. (**C**) Western blot analysis of c-Myc, p-Myc, and ITGB8 protein expression in HCT116 cells treated with DT-061 or FTY-720. (**D** and **E**) Intrasplenic injection mouse models treated with DT-061 (SMAP) via gavage at 5 mg/kg on alternate days (*n* = 5 mice per group). Representative images and quantification of liver metastatic lesions 30 days following HCT116 injection (D). Representative H&E staining of metastatic nodules in the liver (E). (**F** to **J**) HCT116 orthotopic implantation mouse models treated with SMAP via gavage at 5 mg/kg on alternate days (*n* = 8 mice per group). Representative images of primary tumors and liver metastases (F). Quantitative analysis of liver metastasis incidence (G), primary tumor volume and weight (H), number of metastatic nodules (I), and Ki67 IHC staining in primary tumors (J). (**K** to **N**) CT26 orthotopic implantation models treated with SMAP via gavage at 5 mg/kg on alternate days (*n* = 8 to 10 mice per group). Representative images of liver metastases (K). Quantitative analysis of liver metastasis incidence (L), primary tumor volume (M), and number of metastatic nodules (N). Data in (B), (D), (H), (I), (J), (M), and (N) are presented as mean ± SD, with unpaired Student’s *t* test. *P < 0.05, **P < 0.01, and ***P < 0.001.

To evaluate the therapeutic potential of PP2A activation, we used DT-061 [a small-molecule activator of PP2A (SMAP)], which stabilizes the B56α-PP2A holoenzyme in its active state to dephosphorylate c-Myc (*22*). DT-061 treatment reduced c-Myc and ITGB8 protein levels and suppressed the migratory and colonization ability of AKR1B10-deficient CRC/GC cells. We observed similar effects with FTY-720, another PP2A activator that targets the endogenous PP2A inhibitor SET (Fig. 7, A to C, and fig. S8, A to H). Conversely, pharmacological inhibition of PP2A with LB-100 at subinhibitory concentrations robustly reversed c-Myc signaling in control groups, with less pronounced effects in sh*AKR1B10* CRC cells, underscoring PP2A’s role in regulating c-Myc–driven oncogenic plasticity, particularly under AKR1B10-deficient conditions (fig. S8, I to L). In an HCT116 intrasplenic liver metastasis model, DT-061 treatment markedly suppressed liver metastasis induced by AKR1B10 depletion (Fig. 7, D and E). In an orthotopic CRC model, mice implanted with HCT116-sh*AKR1B10* cells in the caecum exhibited more and larger hepatic metastases, compared to the controls, and DT-061 treatment reduced both the number and size of metastases (Fig. 7, F to J, and fig. S9A). However, DT-061 did not significantly affect the size or weight of orthotopic primary tumors, although it modestly reduced Ki67 expression (Fig. 7, H and J, and fig. S9B), suggesting limited antitumor efficacy in primary lesions. In addition, knocking down Akr1b8 promoted hepatic metastases in the CT26 syngeneic orthotopic CRC model. Conversely, DT-061 treatment effectively reduced the incidence of liver metastasis in this system (Fig. 7, K to N, and fig. S9, C to E). These data underscore the critical role of PP2A signaling in AKR1B10-mediated metastasis and highlight the potential of a pharmacological PP2A activator as a therapeutic strategy against AKR1B10-deficient CRC metastasis.

### c-Myc represses *AKR1B10* transcription in gastrointestinal cancer cells

**Fig. 8.**
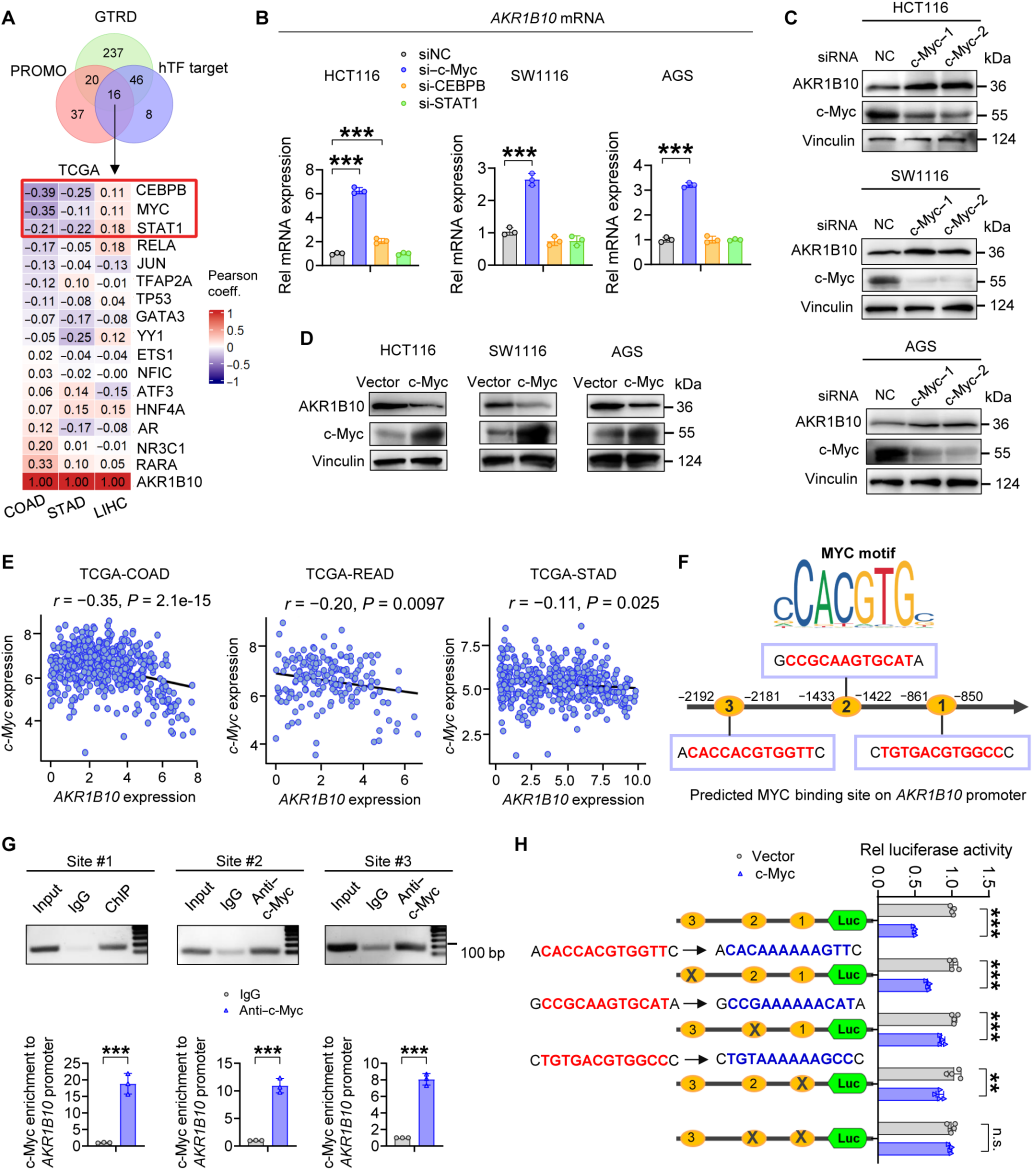
c-Myc suppresses AKR1B10 transcription in gastrointestinal cancers. (**A**) Bioinformatics analysis identifying three putative TFs that regulate AKR1B10 expression and show an inverse coexpression pattern with AKR1B10 between gastrointestinal cancer and HCC, based on TCGA database. coeff., coefficient. (**B**) Quantitative reverse transcription (qRT)–PCR analysis of AKR1B10 mRNA expression in HCT116, SW1116, and AGS cells transfected with small interfering RNAs (siRNAs) targeting c-Myc, CEBPB, or STAT1. (**C** and **D**) Western blot analysis of AKR1B10 protein expression in HCT116, SW1116, and AGS cells after c-Myc knockdown (C) or overexpression (D). (**E**) Correlation between AKR1B10 and c-Myc mRNA levels in human COAD (*n* = 494), READ (*n* = 173), and stomach adenocarcinoma (STAD; *n* = 405) tissues, based on TCGA database. (**F**) Identification of putative c-Myc binding sites within the *AKR1B10* gene promoter region using the JASPAR database. (**G**) ChIP-qPCR analysis depicting the binding sites of c-Myc within the *AKR1B10* promoter region in HCT116 cells. (**H**) Dual-luciferase reporter assay showing that mutations at both site 1 (−850 ~ −861) and site 2 (−1422 ~ −1433) abrogated c-Myc–mediated repression of *AKR1B10* promoter activity. Luc, luciferase. Data in (B), (G), and (H) are presented as mean ± SD (*n* = 3), with unpaired Student’s *t* test. ***P < 0.001.

To investigate the mechanism underlying the reduced *AKR1B10* mRNA expression in CRC, especially its opposing expression pattern in gastrointestinal cancer versus HCC, we performed TF prediction analyses using PROMO, hTFtarget, and Gene Transcription Regulation Database (GTRD) databases. Among the identified candidates, CEBPB, c-Myc, and signal transducer and activator of transcription 1 (STAT1) exhibited an inverse coexpression pattern with AKR1B10 in gastrointestinal cancers compared to HCC (Fig. 8A). Functional experiments revealed that c-Myc knockdown increased AKR1B10 expression in CRC and GC cells, whereas silencing CEBPB or STAT1 resulted in minor or negligible effects (Fig. 8, B and C, and fig. S10, A to C). Overexpression of exogenous c-Myc reduced AKR1B10 protein levels in gastrointestinal cancer cells (Fig. 8D). Analysis of TCGA datasets consistently revealed a robust negative correlation between c-Myc and AKR1B10 expression in colon [correlation coefficient (*r*) = −0.35], rectal (*r* = −0.2), and stomach adenocarcinoma (*r* = −0.11) samples (Fig. 8E). By comparison, a modest positive correlation is evident in liver HCC (*r* = 0.11), with stronger positive correlations in lung adenocarcinoma (*r* = 0.18) and lung squamous cell carcinoma (*r* = 0.36) (fig. S10D). Bioinformatics revealed three putative c-Myc binding sites within the *AKR1B10* promoter region (Fig. 8F). ChIP confirmed c-Myc enrichment at these regions with different binding affinity (Fig. 8G). Mutating sites −850 ~ −861 and −1422 ~ −1433 together abolished c-Myc–mediated suppression of *AKR1B10* promoter activity, as shown in luciferase reporter assays (Fig. 8H). c-Myc knockdown in HCC cell lines (MHCC97-H and Huh7) exerted negligible effects on AKR1B10 expression at either protein or transcriptional level, implying a cancer type–specific regulatory mechanism (fig. S10, E and F). These results identified AKR1B10 as a direct transcriptional target of c-Myc and established a c-Myc–dependent positive feedback loop, contributing to AKR1B10 down-regulation in CRC.

### AKR1B10 is down-regulated in metastatic hepatic lesions and correlates with CRC aggressiveness

**Fig. 9.**
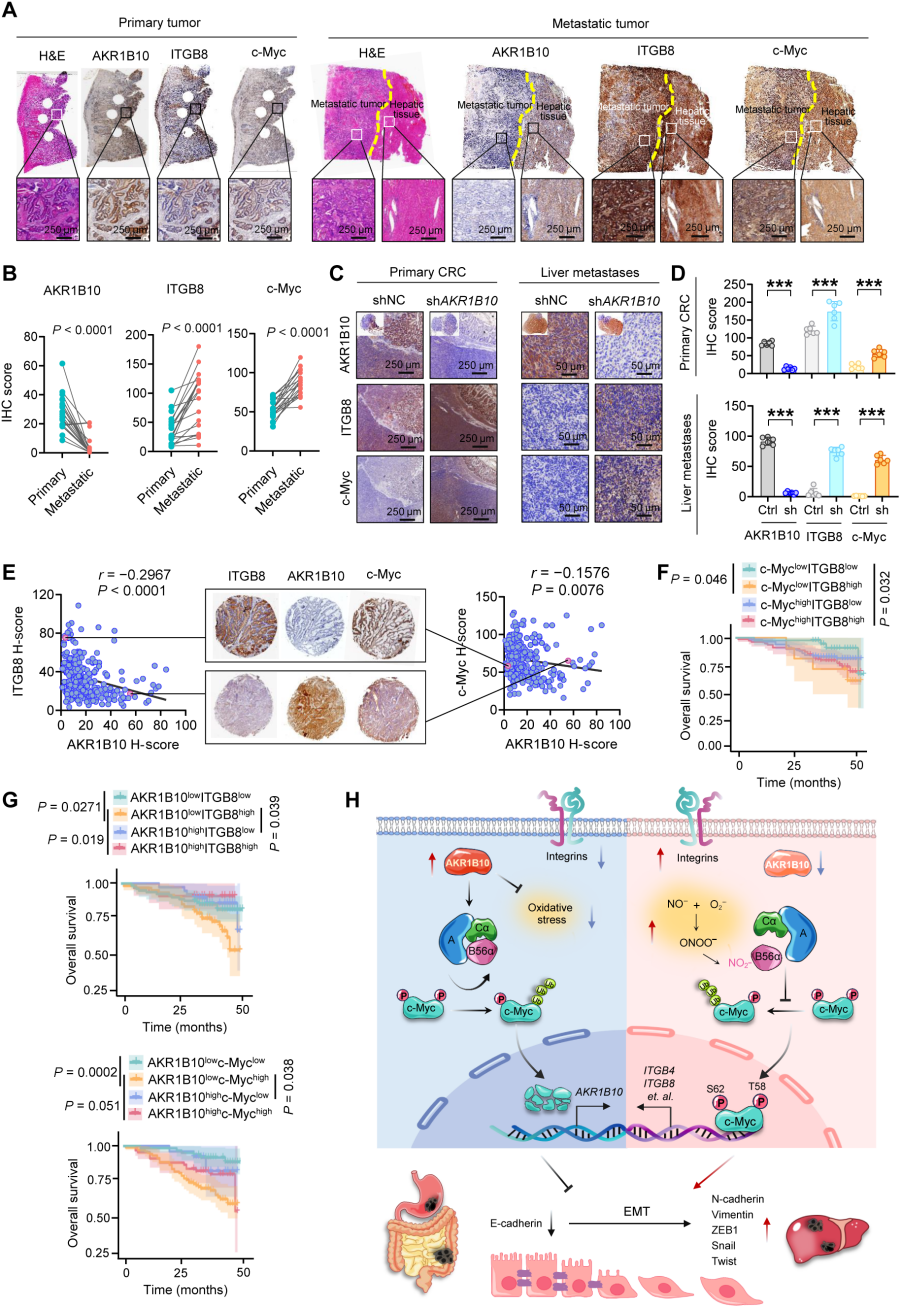
Dysregulation of the AKR1B10–c-Myc–ITGB8 axis drives CRC progression. (**A** and **B**) Representative H&E and IHC images (A) and quantification (B) of AKR1B10, ITGB8, and c-Myc staining in paired primary CRC tissues and liver metastatic lesions from 20 patients. (**C** and **D**) Representative IHC images (C) and histoscore (D) of AKR1B10, ITGB8, and c-Myc staining in primary tumors and liver sections from the HCT116 orthotopic implantation mouse model (*n* = 6). (**E**) Correlation analysis of IHC scores between AKR1B10 and ITGB8 or c-Myc in CRC TMAs. (**F** and **G**) Kaplan-Meier survival curves of patients with CRC stratified by high or low c-Myc/ITGB8 expression levels (F) and AKR1B10/ITGB8 or AKR1B10/c-Myc expression levels (G) from CRC TMAs. (**H**) Schematic diagram illustrating how down-regulated AKR1B10 accelerates CRC metastasis. Ub, ubiquitin. Data are presented as mean ± SD, with paired (B) or unpaired (D) Student’s *t* test or log-rank test (G).

To assess the clinical significance of AKR1B10 in CRC metastasis, we performed IHC analyses to compare expression levels in primary CRC tissues and matched liver metastatic lesions. The AKR1B10 expression was markedly reduced in metastatic lesions, while c-Myc and ITGB8 levels were elevated compared to primary tumors (Fig. 9, A and B). Consistently, in an orthotopic CRC mouse model, AKR1B10 depletion markedly increased c-Myc and ITGB8 expression in both primary tumors and liver metastatic foci compared to controls (Fig. 9, C and D). Correspondingly, TMA analysis of human CRC (hCRC) samples revealed a strong negative correlation between AKR1B10 and both c-Myc and ITGB8 expressions (Fig. 9E). Low AKR1B10 expression, especially in combination with high c-Myc or ITGB8 expression, strongly predicted poor prognosis in patients with CRC based on TMA analysis. Notably, concurrent high expressions of both c-Myc and ITGB8 correlated with unfavorable clinical outcomes. Patients with c-Myc^low^ITGB8^high^ tumors exhibited worse prognosis compared to those with c-Myc^low^ITGB8^low^ tumors, underscoring the critical oncogenic roles of c-Myc and ITGB8 in CRC progression (Fig. 9, F and G). Together, these findings underscore the clinical relevance and prognostic value of AKR1B10, with key mechanistic insights into the AKR1B10–c-Myc–integrin axis in CRC metastasis.

## DISCUSSION

This study was motivated by the observed aberrant down-regulation of AKR1B10 in gastrointestinal cancers and its enigmatic role in disease progression. We demonstrate that AKR1B10 acts as a crucial suppressor of CRC metastasis by functioning as a bona fide endogenous activator of the PP2A holoenzyme through its redox enzymatic activity and direct interaction with PP2Ac. Loss of AKR1B10 stabilizes c-Myc via sustained S62 phosphorylation, promoting integrin transcription and EMT. Notably, the phospho-stabilized c-Myc transcriptionally represses AKR1B10, creating a positive feedback loop that amplifies its reduction in CRC. L125 of AKR1B10 is essential for PP2Ac binding and redox-regulated B56α nitration (Fig. 9H). Pharmacologically restoring PP2A activity may offer a promising therapeutic avenue to counter AKR1B10 down-regulation–driven metastasis. This study highlights AKR1B10 as a critical nexus between the redox milieu and metastasis, advancing understanding of redox-regulated posttranslational modification in fine-tuning the PP2A–c-Myc axis and offering insights into antimetastatic strategies.

The contrasting expression patterns of AKR1B10 between gastrointestinal cancers and nondigestive tract malignancies highlight its dichotomic role in tumorigenesis. While AKR1B10 promotes tumorigenesis in HCC, breast cancer, lung cancer, and pancreatic cancer (*41*, *48*–*50*), we demonstrate that its expression predicts better prognosis in gastrointestinal cancers, based on analyses of public databases and patient samples from FAH-SYSU. Our findings link AKR1B10 down-regulation to c-Myc–ITGB8 activation, which drives EMT and facilitates secondary organ colonization. This conclusion is further supported by their inverse correlation in TMAs and metastatic samples derived from patients with CRC, underscoring AKR1B10–c-Myc–integrin signaling in governing inherent plasticity of CRC.

AKR1B10 is known to exert oncogenic functions in nondigestive solid tumors by promoting fatty acid synthesis, activating KRAS via protein prenylation, and enhancing metabolic adaptability (*35*, *41*, *51*). Our findings reveal a contrasting role for AKR1B10 as a tumor suppressor in CRC metastasis, mediated through posttranslational activation of PP2A. While AKR1B10 depletion exerted marginal effects on the glycolytic capacity of CRC cells, it suppressed gastrointestinal cancer metastasis through its reductive enzymatic activity targeting retinal and reactive aldehydes. However, its influence on other metabolic pathways, such as fatty acid synthesis and lipid metabolism, and their potential impact on CRC progression need to be clarified. In addition, as a secretory protein pivotal in managing inflammatory signaling pathways (*39*), AKR1B10’s paracrine effects within the tumor microenvironment and its immunomodulatory roles in tumor progression warrant further elaboration.

Deregulated redox homeostasis is a hallmark of cancer cells, driving tumor growth, promoting angiogenesis, and enhancing invasiveness and metastatic potential (*52*, *53*). As an NADPH-dependent oxidoreductase, AKR1B10 plays a crucial role in redox regulation through its enzymatic activity. It efficiently reduces reactive aldehydes like 4-hydroxynonenal, a byproduct of lipid peroxidation (*54*). AKR1B10 knockdown elevates cellular carbonyl levels, exacerbating ROS accumulation and oxidative stress in CRC and HCC cells (*48*, *55*). In this study, we observed that AKR1B10 depletion increased ROS production, which serves as a second messenger through oxidative modification on PP2A heterocomplex. Specifically, ONOO^−^-mediated nitration of PP2A subunits, such as B56α, inhibited holoenzyme assembly, sustaining S62 phosphorylation of c-Myc (*18*, *26*, *56*). We uncovered that AKR1B10 depletion in CRC cells enhanced redox-dependent c-Myc stabilization via PP2A-B56α nitration, pinpointing its central role in redox-modulated c-Myc activation. Notably, AKR1B10 and PP2A synergistically regulate c-Myc signaling—enhancement of either suppresses it, while inhibition amplifies it (Fig. 5, N and O, and fig. S8, I to L). These findings suggest that AKR1B10 also modulates the c-Myc–ITGB8 axis through a minor PP2A-independent mechanism. Further exploration is required to determine whether AKR1B10 affects redox modification of other PP2A subunits and their downstream substrates. Expanding this research to proteins sensitive to tyrosine nitration, such as p53, procaspase-3, and Bcl-2 (*18*, *57*, *58*), may uncover broader mechanisms by which AKR1B10 governs redox-dependent signaling in cancer.

AKR1B10 also displays noncanonical functions independent of its enzymatic activity. It has been shown to interact with HSP90α, which facilitates its lysosomal secretion as a molecular chaperone (*59*). In addition, AKR1B10 binds to acetyl–coenzyme A carboxylase α, a rate-limiting enzyme in de novo fatty acid synthesis, preventing its ubiquitination and degradation (*51*). In this study, we demonstrate that AKR1B10 promotes PP2A holoenzyme assembly by directly interacting with PP2Ac, serving as an endogenous activator of PP2A through both its reductase activity and moonlighting function. Beyond regulating retinal reductase activity, we find the critical role of K125 in mediating B56α nitration and facilitating AKR1B10-PP2Ac interaction. Notably, the AKR1B10^K125L^ mutant amplified c-Myc’s oncogenic effects and promoted CRC metastasis by suppressing PP2A activity. Our study emphasizes the clinical relevance of AKR1B10 as both a diagnostic biomarker and a molecular target for tailored therapeutic strategies aimed at alleviating c-Myc–driven metastasis.

PP2A is a well-established tumor suppressor, frequently subject to genetic alterations or functional inactivation, contributing to the development of various human malignancies (*60*–*64*). Its enzymatic activity is finely regulated by posttranslational modifications, including phosphorylation, nitration, and methylation (*18*, *24*–*26*). In this study, we present the first evidence that AKR1B10 acts as a precise regulator of PP2A activity. Moreover, we showed that pharmacological restoration of PP2A activity efficiently mitigates the metastatic potential induced by AKR1B10 loss during CRC progression. Our findings highlight the feasibility of repurposing PP2A activators to counteract AKR1B10–PP2A–c-Myc axis–mediated CRC metastasis.

In summary, this study uncovers the tumor-suppressive attributes of the aldo-keto reductase AKR1B10 in CRC (phenocopies in GC), where it unleashes PP2A activity through both its redox regulatory capacity and moonlighting, binding-dependent function. We propose pharmacological restoration of PP2A activity as a promising strategy to counteract the prometastatic effects mediated by AKR1B10 depletion. Given AKR1B10’s contrasting expression patterns across various malignancies, these findings provide valuable insights into the intricate mechanisms of AKR1B10 dysregulation in tumorigenesis, paving the way for promising therapeutic strategies.

## MATERIALS AND METHODS

### Cell culture

hCRC cells [HCT116 (Dukes’ stage D, mesenchymal subtype), SW1116 (Dukes’ stage A, canonical epithelial subtype), LoVo (Dukes’ stage C, immune-activated subtype), and HT29 and SW620], human GC (hGC) cells (MKN45, AGS, HGC27, and MKN74), human gastric epithelial cells (GES-1), mouse CRC cells (MC38 and CT26), human HCC cells (MHCC97-H and Huh7), and human embryonic kidney (HEK) 293T cells were obtained from the American Type Culture Collection (Manassas, VA, USA) or maintained in our laboratory. Cells were cultured at 37°C with 5% CO_2_ in a humidified incubator with Dulbecco’s modified Eagle’s medium (DMEM) or RPMI 1640 medium supplemented with 10% fetal bovine serum (FBS), penicillin/streptomycin (100 U/ml), and 1 mM sodium pyruvate. All cell lines were routinely checked using the short tandem repeat method and tested negative for mycoplasma.

### Clinical specimens

Human tissue specimens, including TMAs containing paired CRC and adjacent normal tissues (between December 2019 and December 2021), 93 CRC tissues for IHC staining, RNA and protein samples from matched CRC/GC tissues and adjacent normal tissues, and 20 paired CRC primary tumor samples and metastatic liver samples were obtained from the SYSU-FAH with the consent of the patients. Patients with CRC were stratified into four groups based on the expression combinations of AKR1B10-ITGB8, AKR1B10–c-Myc, and c-Myc–ITGB8, categorized as low (H-score < 40) or high (H-score ≥ 40). Kaplan-Meier curves were generated to evaluate overall survival among the four groups. This study was approved by the Medical Ethics Committee, SYSU-FAH ([2024]476), with the consent of the patients. The clinicopathological characteristics of the patients with CRC whose samples were included in TMAs are summarized in table S1.

### Plasmid construction and transfection

pCMV-HA-PPP2CA, pCMV-3×Flag-AKR1B10, and pCMV-His-PPP2R5A were obtained from MiaolingBio (Wuhan, China). Mutant constructs of AKR1B10 (K125L, V301L, C299S, P219S, and K263R) and PPP2R5A (Y238F) were generated by site-directed mutagenesis and cloned into the pCMV-3×Flag and pcDNA4/myc-His A vectors, respectively. The primers used in the site-directed mutagenesis are listed in table S2. To generate reconstituted cell lines in hCRC-sh*AKR1B10* or hGC-sh*AKR1B10*, cells were transfected with pCMV-3×Flag-AKR1B10, pCMV-3×Flag-AKR1B10^K125L^, pCMV-3×Flag-AKR1B10^V301L^, or pCMV-3×Flag-AKR1B10^C299S^ using Lipofectamine 3000 (Invitrogen, L3000008). The stable cell lines were selected with G418 (Sigma-Aldrich, A1720) and confirmed by quantitative polymerase chain reaction (qPCR) and Western blot. For small interfering RNA (siRNA)–mediated ITGB8, c-Myc, MAX, RUVBL2, STAT1, and CEBPB silencing, cells were transfected with siRNA duplex and Lipofectamine RNAiMAX (Invitrogen, 13778075) according to the manufacturer’s instructions. A nonspecific siRNA oligo (Sigma-Aldrich, SIC002) was used as a negative control. To establish stable *AKR1B10/Akr1b8* knockdown cell lines, shRNAs targeting the genomic sequences of AKR1B10 and Akr1b8 were inserted into pLKO.1-puro (Addgene) empty vector. Lentiviral particles were produced by transfecting HEK293T cells with pMD2.G, psPAX2, and pLKO.1-shRNA plasmids at a 1:2:2 ratio using Lipo8000 reagent (Beyotime, C0533) for 48 hours. Parental cells were then infected with shRNA-containing lentivirus for 24 hours and selected with puromycin (Selleck, S7417) for 1 to 2 weeks. The sequences of shRNA and siRNA are listed in table S3.

### RNA extraction and quantitative PCR

Total RNA was extracted using a FastPure Cell/Tissue Total RNA Isolation Kit (Vazyme, RC112) according to the manufacturer’s instructions and reversely transcribed into cDNA using the Evo M-MLV RT Kit (Accurate Biology, AG11706). Real-time PCR was performed on a QuantStudio 5 (Applied Biosystems) using the Premix Pro Taq HS qPCR Kit II (Accurate Biology, AG11702). The primers used for quantitative reverse transcription (qRT)–PCR were synthesized by TSINGKE Biotechnology, and their sequences are detailed in table S4.

### RNA-seq and analysis

Total RNA was extracted from HCT116 cells [negative control shRNA (shNC) versus sh*AKR1B10*] using TRIzol reagent (Invitrogen, 15596026). RNA quality was evaluated using the Agilent 2100 Bioanalyzer. Only samples that exhibited an RNA integrity number of 7 or higher were deemed acceptable for further procedures. Subsequent RNA-seq was performed on the Illumina NovaSeq 6000 sequencing machine (BGI Genomics, China). Differential genes (DEGs) were selected using the DESeq2 R package with the following requirements: fold change > 1 and adjusted *P* value < 0.05. The KEGG pathway analysis was performed to annotate the biological function of the DEGs using the clusterProfiler R package. The heatmap of differential gene clusters was generated using the pheatmap function on the differential gene set.

### ChIP assay

ChIP assays were performed using the EZ-ChIP kit (Merck Millipore, 17-375) according to the manufacturer’s instructions. Briefly, HCT116 cells were harvested after crosslinking in 1% formaldehyde at room temperature for 15 min, followed by quenching with 0.125 M glycine (Sigma-Aldrich, V900144) for 5 min. Cells were then washed twice with ice-cold phosphate-buffered saline (PBS), scraped, and pelleted by centrifugation. The cell pellet was resuspended in lysis buffer and subjected to freeze-thaw cycles via liquid nitrogen. Cell lysate was centrifuged to isolate nuclei, which were subsequently resuspended in a diluted enzymatic cocktail to enzymatically shear genomic DNA into fragments ranging from 100 to 200 base pairs (bp). Cleaved chromatin was incubated with 5 μg of anti-Myc antibody (Abcam, ab32072) or only Protein A Agarose/Salmon Sperm DNA. Following washes and decrosslinking, qPCR and agarose gel electrophoresis assays were performed to detect the enriched regions of c-Myc within the AKR1B10 promoter. Primers used for ChIP are listed in table S5.

### Cell viability and colony formation assay

Cells (1 × 10^4^) were seeded in a 96-well plate for cell proliferation assay. Cell viability was measured at 24, 48, 96, and 120 hours using the Cell Counting Kit-8 [MedChemExpress (MCE), HY-K0301]. For the colony-forming assay, the indicated number of cells was seeded in a six-well plate for 12 to 14 days. The colonies were fixed using 4% paraformaldehyde (Biosharp, BL539A-1), stained with crystal violet staining solution (Beyotime, C0121), and counted with the ImageJ v1.53c software. The experiment was performed in three biological replicates.

### Cell migration assays

The transwell assay was performed using polycarbonate membrane chambers with an 8-μm pore (Corning, 3422). Cells (1 to 1.5 × 10^5^) were seeded into the upper transwell chamber with 300 μl of serum-free medium for a 1- to 2-day migration period. The lower chamber was filled with a 500-μl medium containing 20% FBS as a chemoattractant. After 24 to 72 hours of migration, cells that migrated to the lower side were fixed by 4% paraformaldehyde and stained with crystal violet, photographed, and counted. The results of the experiment were determined by three replications.

For the wound healing assay, 3 × 10^5^ cells were inoculated in a six-well plate, and the cell layer was scraped with a sterilized 10-μl pipette tip when the cells reached 90% confluence. Subsequently, the cells were cultured in serum-free medium, and images were captured by an inverted microscope at 0, 12, and 24 hours. ImageJ v1.53c software was used to calculate the wound healing areas.

### Cell cycle and cell apoptosis assay

Cell cycle analysis was performed using the Propidium Iodide Cell Cycle Detection Kit (4A Biotech, FXP0211) according to the manufacturer’s instructions. Briefly, cells were collected and fixed with 70% ethanol at 4°C overnight. Subsequently, the cells were incubated with propidium iodide (PI) staining solution at 37°C in the dark for 30 min. Following three successive washes with PBS, the cells were analyzed using a flow cytometer CytoFLEX (Beckman Coulter). For cell apoptosis analysis, cultured cells were washed, dissociated, and suspended in 1× binding buffer. The cells were then stained with annexin V and PI (4A Biotech, FXP023) at room temperature for 15 min in the dark, followed by washing and further flow cytometry analysis.

### H&E and immunohistochemistry

IHC assays were conducted as previously described (*65*). Paraffin-embedded tumor tissue sections were deparaffinized in xylene and rehydrated with graded ethanol. Postrehydration, the sections were subjected to H&E staining. For IHC staining, the sections underwent rehydration, after which endogenous peroxidase activity was quenched, and heat-induced epitope retrieval was performed. After blocking with normal bovine serum albumin (BSA) antigen for 1 hour at 37°C, the tissue sections were incubated with anti-AKR1B10 (Signalway Antibody, 36071), anti-ITGB8 (Proteintech, 29775-1-AP), or anti–c-Myc (Abcam, ab32072) antibodies at 4°C overnight. Following incubation with horseradish peroxidase (HRP)–conjugated secondary antibodies, the sections were visualized with DAB Detection Kit (ZSGB-BIO, ZLI-9017) and then dehydrated and stabilized with mounting medium, and the images were acquired with a KF-PRO-020 scanner (Konfoong Tech). The specificity of antibodies used for IHC was validated using standard approaches, as demonstrated in fig. S11. IHC analysis was conducted by two independent observers who were blinded to the results of the other markers and clinical outcomes. Staining intensity was categorized as 0 (no staining), 1+ (weak staining), 2+ (moderate staining), or 3+ (intense staining). Multiplying the staining intensity by the reaction extension value (range 0 to 300) yielded the H-score for the tumor tissue.

### Immunofluorescence

Cells seeded on 15-mm glass-bottomed cell culture dishes (Nest, 801002) were fixed with 4% paraformaldehyde for 10 min and permeabilized with 0.5% Triton-X 100 (in PBS) for 10 min at room temperature, followed by blocking, overnight incubation with primary antibody [AKR1B10 mouse monoclonal antibody (1:300; Santa Cruz Biotechnology, sc-365689) and PPP2CA rabbit polyclonal antibody (1:500; ABclonal A6702)], and incubation with secondary antibodies [Alexa Fluor 555–labeled Donkey anti-Rabbit IgG (immunoglobulin G) (1:500, Beyotime, A0453) and Alexa Fluor 488–labeled Goat anti-Mouse IgG (1:500, Beyotime, A0428)] for 1 hour in the dark. Confocal microscopy images were captured using an Olympus FV3000 Laser Scanning Confocal Microscope and evaluated with FV31S-SW software.

### Western blot and immunoprecipitation

Cells were harvested and lysed with radioimmunoprecipitation assay buffer (Beyotime, P0013B) or immunoprecipitation (IP) lysis buffer (Beyotime, P0013). The cell lysates underwent sonication for 10 cycles and were centrifuged at ×12,000 rpm for 10 min. Supernatants were collected, and protein concentrations were determined by bicinchoninic acid (BCA) protein assay kit (Beyotime, P0011). Protein lysates were separated by SDS–polyacrylamide gel electrophoresis (SDS-PAGE) and transferred to polyvinylidene difluoride membranes (Merck Millipore, IPVH00010). Membranes were blocked with 5% nonfat milk in 1 × PBS–Tween 20 for 1 hour at room temperature and probed overnight at 4°C with specific antibodies. The immunoblots were visualized using Immobilon Western HRP substrate (Merck Millipore, WBKLS0500) and scanned using an Amersham ImageQuant 800 system (Cytiva). To determine the half-life of c-Myc, shNC and sh*AKR1B10* CRC/GC cells were exposed to CHX (Sigma-Aldrich, C7698) for varying durations (0, 2, 4, 8, 16, and 24 hours) and subsequently analyzed through Western blot.

After BCA quantification, equal amounts of protein lysates for Co-IP were subjected to a preclearing step using 25 μl of Protein A/G PLUS-Agarose (Santa Cruz Biotechnology, sc-2002) for 1 hour on ice and then incubated with the primary antibodies and Protein A/G PLUS-Agarose on a rocker platform at 4°C overnight. Immunoprecipitates were collected by centrifugation at 2000 rpm for 5 min at 4°C, washed thrice with cold lysis buffer, and eluted by boiling in 1 × SDS-PAGE loading buffer for 6 min and then subjected to Western blot analysis. Antibodies used for Western blot and IP are listed in table S6. The specificity of antibodies targeting c-Myc and pT58 c-Myc was validated in fig. S11.

### Purified protein pull-down assay

The pull-down protein-protein interaction assay was performed as previously described (*14*). Glutathione-Sepharose slurry (Cytiva, 17075601) beads were equilibrated by incubating for 3 × 10 min in PBS buffer at 4°C. Following equilibration, 3 μg of recombinant GST-AKR1B10 protein (CusaBio, CSB-EP001540HU) or GST-AKR1B10 (K125L) protein [CusaBio, CSB-EP001540HU(M)] was incubated with the beads at room temperature for 1 hour. Then, 3 μg of recombinant His-PPP2CA (CusaBio, CSB-EP018559HUc7) or His-PPP2R1A (CusaBio, CSB-EP018562HUa0) was added to the buffer containing protein-bound glutathione beads overnight at 4°C. After three washes with PBS, the bead-bound complex was resuspended in SDS loading dye and boiled at 95°C. Eluted proteins were subjected to SDS-PAGE and immunoblotted with indicated antibodies.

### Flow cytometry analysis

The DCFH-DA Assay Kit was used for the detection of cellular ROS. Cells were plated in six-well culture dishes at a density of 1 × 10^5^ cells per well and treated with DPI (Selleck, S8774) or FeTPPS (MCE, HY-131697) for indicated durations. Following treatment, cells were trypsinized, centrifuged, and resuspended in PBS. They were then incubated in the dark at 37°C for 30 min with 5 μM DCFH-DA probe (MCE, HY-D0940). Following three successive washes with PBS, the abundance of ROS was detected using a flow cytometer CytoFLEX (Beckman Coulter), and the data were processed via FlowJo 10.8.1.

### Luciferase reporter assay

The h*AKR1B10* promoter regions, spanning from −2500 to +1 of exon 1, were cloned into the pGL3-Basic vector. Luciferase activities were evaluated using the Dual-Luciferase Reporter Assay System (Promega, E1910) according to the manufacturer’s instructions. HCT116 cells were seeded into a 12-well plate and transiently transfected with WT or mutant luciferase reporter plasmids plus Renilla DNA (pRL-TK) at a ratio of 10:1, after transfection with control or HA-MYC plasmid. HCC cells (MHCC97-H and Huh7) were seeded into a 12-well plate and transiently transfected with WT luciferase reporter plasmids plus Renilla DNA (pRL-TK) at a ratio of 10:1, after transfection with negative control siRNA or si–c-Myc RNAs. At 24 hours posttransfection, cells were washed with PBS, lysed with 1× passive lysis buffer, and transferred into a white 96-well plate for bioluminescence detection. Firefly luciferase activities were normalized by Renilla luciferase signal.

### PP2A activity assay

PP2A activity was measured by the Serine/Threonine Phosphatase Assay Kit (Promega, V2460). Briefly, cells were collected and lysed by phosphatase storage buffer [20 mM imidazole-HCl, 2 mM EDTA, 2 mM EGTA (pH 7.0) with 10 μg/ml each of aprotinin, leupeptin, antipain, soybean trypsin inhibitor, 1 mM benzamidine, and 1 mM phenylmethylsulfonyl fluoride]. Lysates were then sonicated for 3 × 1.5 s, centrifuged at 13,000 rpm at 4°C for 30 min, and added to Sephadex G-25 spin columns to remove endogenous free phosphate. Following protein quantification by BCA Protein Assay Kit, protein lysates (500 μg) were incubated with 5 μg of antibody anti-PPP2R5A (12675-2-AP) (*66*) and 50 μl of Protein A/G PLUS-Agarose at 4°C overnight with constant rocking. A total of 5 μl of phosphopeptide RRA(pT)VA (1 mM) and 10 μl of PP2A reaction buffer [250 mM imidazole (pH 7.2), 1 mM EGTA, 0.1% β-mercaptoethanol, and BSA (0.5 mg/ml)] were premixed and added to each 96-well plate for preincubation at 37°C. The beads were washed three times with tris-buffered saline and resuspended in 150 μl of phosphatase storage buffer. The reaction was started by adding the phosphatase samples (35 μl) to the wells containing the premixed reaction solution, and the reactions were incubated for 30 min. The reaction was stopped by adding 50 μl of molybdate dye/additive mixture. Following 30-min incubation, absorbance was measured at 630 nm in a 96-well microplate reader (Varioskan LUX).

### Tumor spheroid culture

HCT116 or SW1116 cells were cultivated in DMEM or RPMI 1640 medium, respectively, supplemented with 10% (v/v) FBS and 2.5% (v/v) Matrigel (Corning, 356234). A total of 4000 cells per well were seeded into ultralow-attachment 96-well plates (Corning, 7007). Following centrifugation at 1000*g* for 10 min, the cells were cultured for 7 days and then photographed. Images were analyzed using ImageJ.

### Animal experiments

All mice, including BALB/c nude mice, BALB/c mice, C57BL/6 mice, and NOD-SCID mice, were purchased from Guangdong GemPharmatech. All animal experiments were conducted in accordance with the National Institute of Health Guide for the Care and Use of Laboratory Animals. The animals were maintained under specific pathogen–free conditions. The experimental procedures were approved by the Institutional Animal Care and Use Committee of SYSU (approval numbers: SYSU-IACUC-2022003193 and SYSU-IACUC-2023355).

For the subcutaneous xenograft model, 4- to 6-week-old female BALB/c nude mice were randomly allocated to two groups. HCT116 cells (3 × 10^6^; shNC&sh*AKR1B10*) were subcutaneously injected into the dorsal flank of BALB/c nude mice. Tumor volume was measured every 3 days since day 7 of tumor implantation and calculated using the formula *V* = 0.5 × *D* × *W*^2^ (*V* for volume, *D* for diameter, and *W* for width). The mice were euthanized on day 25, and the tumors were photographed and weighed.

For the metastasis model, 4- to 6-week-old female BALB/c nude mice were randomly allocated to two groups. HCT116 cells (1 × 10^6^) were administered through intravenous injection to establish a pulmonary metastasis model or intrasplenic injection to establish a liver metastasis model. Three to four weeks after the injection, the mice were euthanized, and the lungs or livers were photographed and paraffin embedded for H&E staining and IHC. For mice receiving drug treatment, after 7 days of intrasplenic injection, mice were orally gavaged with *O*-carboxymethylcellulose (CMC)–Na (negative control) or SMAP (Selleck, S8774) at 5 mg/kg on alternate days for 3 weeks.

MC38 (5 × 10^5^) cells were administered via injection into the spleen of NOD-SCID mice or C57BL/6 mice (4- to 6-week-old; female). After inoculation for 2 to 5 weeks, mice were euthanized, and the metastatic liver specimens were removed, photographed, weighed, paraffin embedded, and lastly validated by H&E staining and IHC.

To establish the orthotopic mouse model, 4- to 6-week-old female BALB/c nude mice or BALB/c mice were randomly allocated to several groups. Mice were anesthetized by intraperitoneal injection, and a small incision was made to exteriorize the caecum. HCT116 cells (2 × 10^6^) or CT26 cells (0.8 × 10^6^) in 50 μl of PBS were injected into the caecum termini of the mouse, followed by the suturing of the incision. After 7 days of injection, mice were orally gavaged with CMC-Na or SMAP at 5 mg/kg on alternate days. Upon the onset of moribundity in one or more mice, all animals were humanely euthanized. Subsequently, the intestinal and liver tissues were excised, photographed, and paraffin embedded.

### MS analysis

HCT116-sh*AKR1B10* cells stably expressing Flag-AKR1B10 or Flag-vector were lysed in IP lysis buffer (Beyotime, P0013) and immunoprecipitated with anti-FLAG antibody (Sigma-Aldrich, F1804) and Protein A/G PLUS-Agarose (Santa Cruz Biotechnology, sc-2003). The immunoprecipitated complexes were eluted by boiling in 1× loading buffer and then subjected to SDS-PAGE. After staining with Coomassie Brilliant Blue (Beyotime, P0017), gel fragments containing proteins were excised, destained, reduced, and subjected to in-gel trypsin digestion overnight. The extracted peptides were redissolved in 0.1% formic acid mobile phase and centrifuged at 20,000*g* for 10 min, and the supernatants were divided by EASY-nLC 1200 System (Thermo Fisher Scientific) and subjected to liquid chromatography–tandem MS (LC-MS/MS) on the Orbitrap Exploris 480 (Thermo Fisher Scientific) by BGI Genomics. For protein identification, spectra were processed using Mascot v2.3 and searched against the Swiss-Prot database (European Bioinformatics Institute). The output was preprocessed and prescored using Percolator for quality control. Peptides (false discovery rate < 0.01) were selected, and the proteins that contain high-quality and unique peptides were considered qualified. The extracted ion current of each peptide was used for the calculation of peak areas. The final intensity-based absolute quantification (iBAQ) value was based on the iBAQ algorithm by dividing the total peak area by the number of theoretically observable peptides.

### Computational molecular docking

The three-dimensional structure data for AKR1B10 (AF-O60218-F1) were obtained from the AlphaFold Protein Structure Database (https://alphafold.ebi.ac.uk/). The structure of PP2A protein [Protein Data Bank (PDB) ID: 6NTS] was retrieved from PDB (www.rcsb.org). The protein-protein docking model was performed using GRAMM (Global RAnge Molecular Matching) (https://gramm.compbio.ku.edu/) algorithm (*67*). From the top 10 ranked models generated, the pose exhibiting the highest docking score (indicating the most favorable binding energy) was selected to predict the interaction interface between AKR1B10 and PPP2CA. Prediction of hydrogen bonding between amino acid residues and structural illustrations was prepared with UCSF Chimera (www.cgl.ucsf.edu/chimera/).

### Bioinformatic analysis

The data on AKR1B10 mRNA expression were extracted from the TCGA database. Pearson correlations between AKR1B10 and integrin family genes or MYC are based on the TCGA data. The survival data were extracted from TCGA database and GEO datasets (GSE39582, GSE17536, and GSE14210). AKR1B10 protein expression was collected from the Proteomic Data Commons and analyzed by cProSite. AKR1B10 expression in different stages from TCGA data was collected from the University of Alabama at Birmingham Cancer data analysis portal.

### Statistical analysis

All in vitro experiments were independently replicated at least three times, and in vivo animal studies were repeated at least twice. The numbers of biological replicates (*n*) are provided in the figure legends. All values shown in graphs are presented as individual data points and the mean ± SD, unless stated otherwise. Statistical significance was performed using GraphPad Prism 9, with Student’s *t* test for two independent groups and one- or two-way analysis of variance (ANOVA) test for multiple groups (≥3). Survival analyses were conducted using the log-rank test, and correlation analyses were performed using Pearson’s correlation coefficient (*P* < 0.05 was considered statistically significant). Researchers blinded to group assignments performed data collection and analysis for animal experiments and IHC staining.
