# Supplementary material for: AKR1B10 dictates c-Myc stability to suppress colorectal cancer metastasis via PP2A nitration
Source: Sci Adv. 2025 Aug 22;11(34):eadv6937. doi: 10.1126/sciadv.adv6937 (PMC12372902; doi:10.1126/sciadv.adv6937)
Supplement: Supplementary file 1 — Figs. S1 to S11 Tables S1 to S6 [file sciadv.adv6937_sm.pdf]

Science Advances
AAAS

Supplementary Materials for

AKR1B10 dictates c-Myc stability to suppress colorectal cancer metastasis via PP2A nitration

Xiaoxue Wu *et al.*

Corresponding author: Xianzhi Liu, liuxzh37@mail2.sysu.edu.cn; Weiling He, wlhe@xah.xmu.edu.cn; Mei Song, songm7@mail.sysu.edu.cn

Sci. Adv. **11**, eadv6937 (2025)
DOI: 10.1126/sciadv.adv6937

This PDF file includes:

Figs. S1 to S11
Tables S1 to S6

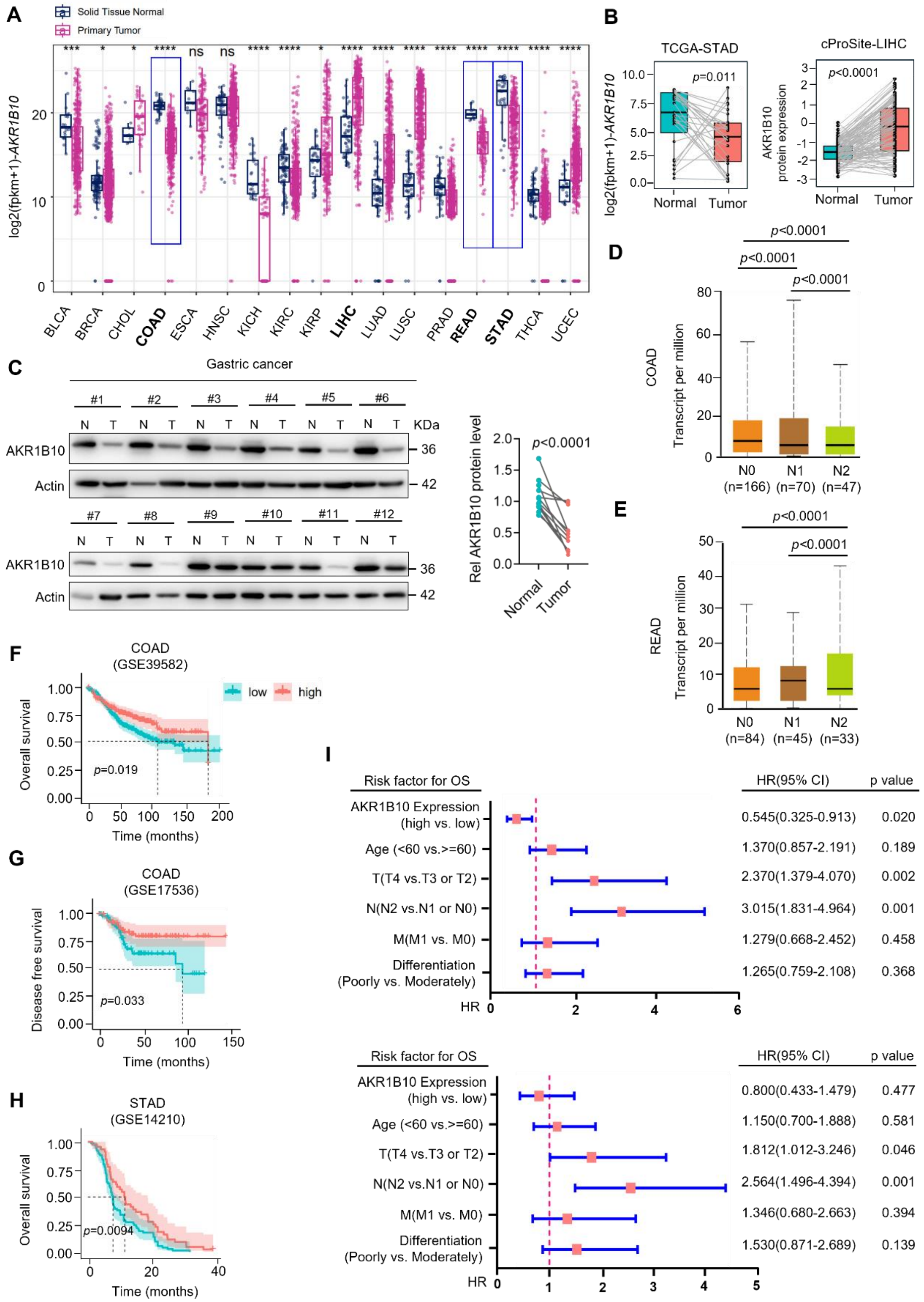

Fig. S1. Low AKR1B10 expression is associated with advanced clinicopathological characteristics in CRC. (**A**) AKR1B10 mRNA levels in various cancers compared to corresponding normal tissues based on TCGA database. (**B**) AKR1B10 expression in stomach

adenocarcinoma (STAD, n=27) or liver hepatocellular carcinoma (LIHC, n=49) tissues and paired normal tissues based on TCGA or cProSite database. (**C**) Western blot analysis of AKR1B10 protein expression in 12 paired adjacent normal tissues (N) and gastric cancer (GC) tissues (T) from SYSU-FAH. (**D** and **E**) The mRNA expression of AKR1B10 divided by the status of lymph node metastasis of patients with COAD (D) or READ (E) from the UALCAN website. (**F** and **H**) Kaplan-Meier plot showing the correlation between AKR1B10 expression and the overall survival rates of COAD patients from GSE39582 (F, n=562) and STAD patients from GSE14210(H, n=119). (**G**) Kaplan-Meier plot showing the correlation between AKR1B10 expression and the disease-free survival rates of COAD patients from GSE17536 (n=177). (**I**) Forest plot showing univariate (upper) and multivariate (lower) Cox regression analysis of different clinical parameters for CRC patients from SYSU-FAH (n=93). HR, hazard ratio; CI, confidence interval. Unpaired Student's *t* test [(A), (D), and (E)], paired Student's *t* test [(B) and (C)], or log-rank test [(F) to (H)].

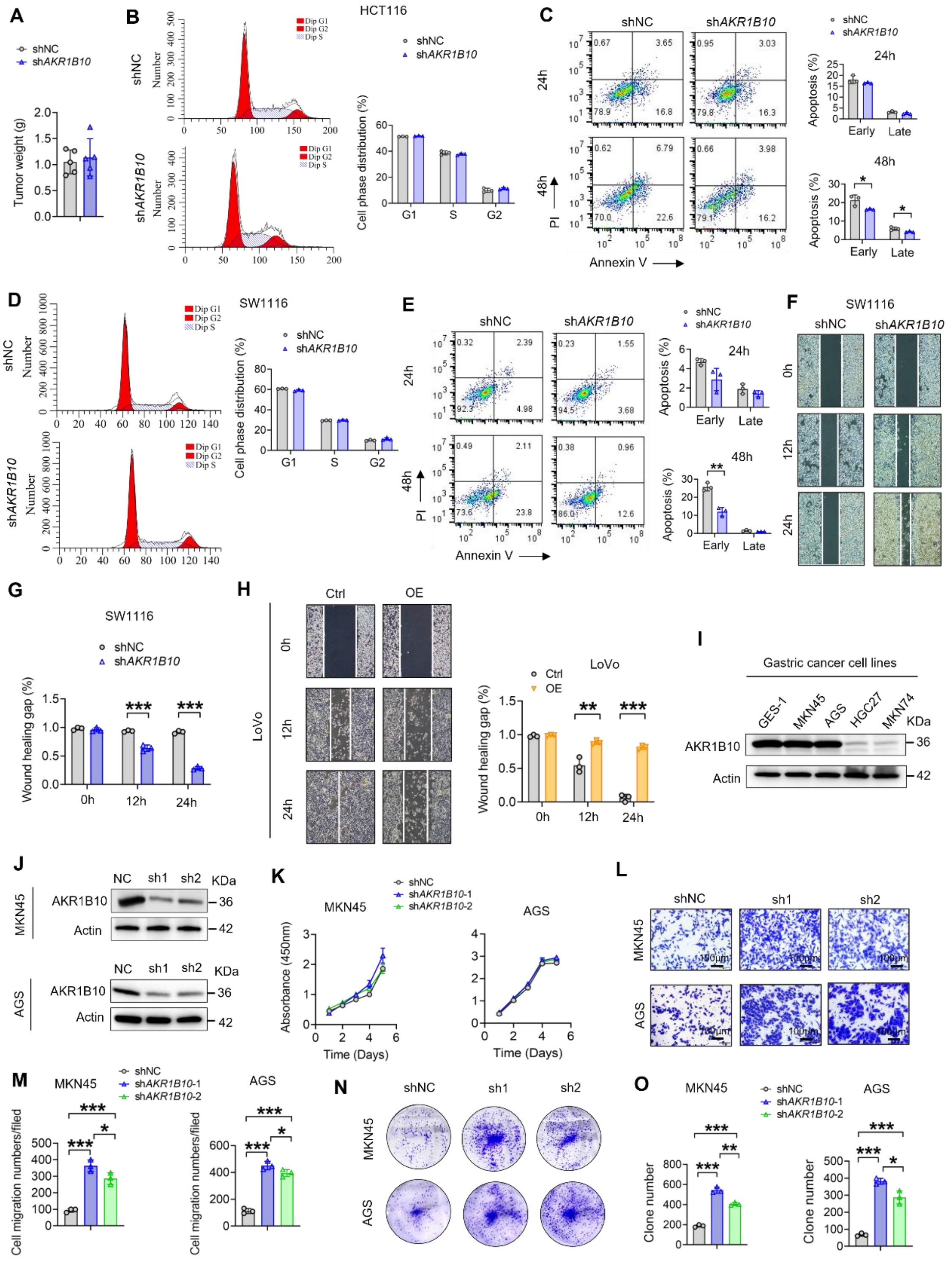

Fig. S2. AKR1B10 suppresses metastasis in CRC and GC. (**A**) Tumor weights of HCT116 (shNC&sh*AKR1B10*) xenografts from nude mice (n=5 mice per group). (**B** and **D**) Cell cycle analysis of HCT116 (B) and SW1116 (D) (shNC&sh*AKR1B10*) 24h after seeding. (**C** and **E**) Apoptosis analysis of HCT116 (C) and SW1116 (E) cells at the indicated time points. (**F-G**) Representative images (F) and quantification (G) of wound-healing assay of SW1116

(shNC&sh*AKR1B10*) cells at indicated time after scratching. (**H**) Representative images and quantification of wound-healing assay of LoVo (Ctrl&AKR1B10-OE) cells at indicated time after scratching. (**I**) Western blot analysis of AKR1B10 protein expression in various human GC cell lines. (**J** and **K**) Validation of AKR1B10 knockdown efficiency in MKN45 and AGS cells by western blot (J) and assessment of cell proliferation with CCK-8 assay at the indicated timepoints (K, n=4). (**L** and **M**) Representative images (L) and quantification (M) of transwell assays in MKN45/AGS cells after 48h. (**N** and **O**) Representative images (N) and quantification (O) of clonogenic assays in MKN45/AGS cells following a 10-14-day incubation period. Mean±SD, unpaired Student's *t* test [(B) to (E), (G), (H), (M), and (O); n=3]. *$p < 0.05$, **$p < 0.01$, ***$p < 0.001$

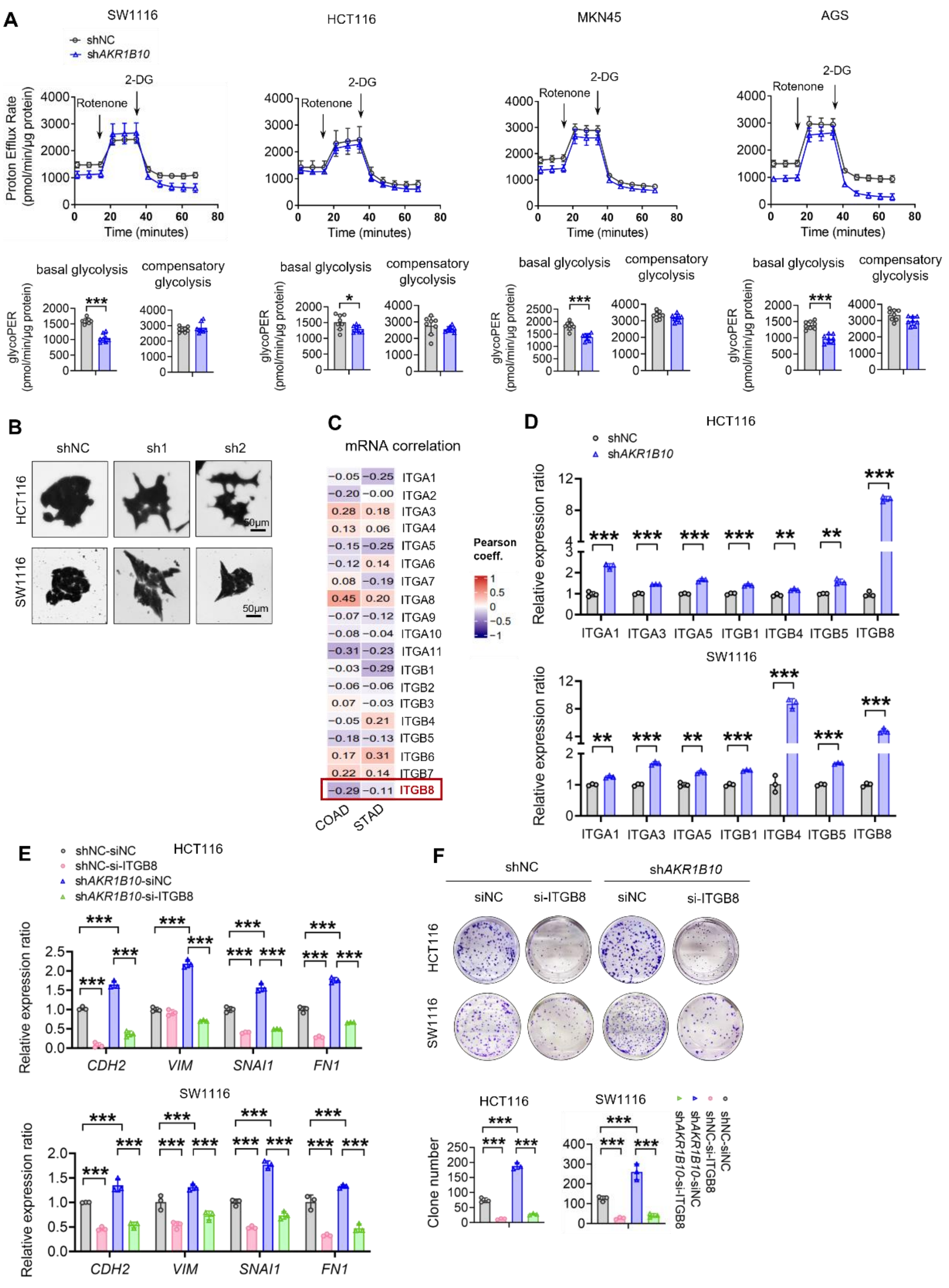

Fig. S3. AKR1B10 inhibits EMT by downregulating integrin signaling in CRC cells. (**A**) Seahorse XF glycolytic rate assay determines proton efflux rate (PER) under basal conditions, after inhibiting oxidative phosphorylation with rotenone, and after inhibiting glycolysis with 2-DG (n=8 replicates per group). Basal glycolysis and compensatory glycolysis were quantified. (**B**) Representative images for cell morphology of HCT116 and SW1116 (shNC&sh*AKR1B10*)

cells. (**C**) Correlation analysis of AKR1B10 with various integrin gene mRNA expressions based on TCGA database ($p<0.05$). (**D**) qPCR analysis of integrin mRNA expression in HCT116 and SW1116 (shNC&sh*AKR1B10*) cells. (**E**) qPCR analysis of the mRNA expression of EMT-related factors in HCT116 and SW1116 cells after ITGB8 silencing. (**F**) Representative images and quantification of clonogenic assays in HCT116 and SW1116 cells after ITGB8 silencing. [(D) to (F)] n = 3. Mean±SD, unpaired Student's *t* test [(A), (D), (E), and (F)]. **$p < 0.01$, ***$p < 0.001$

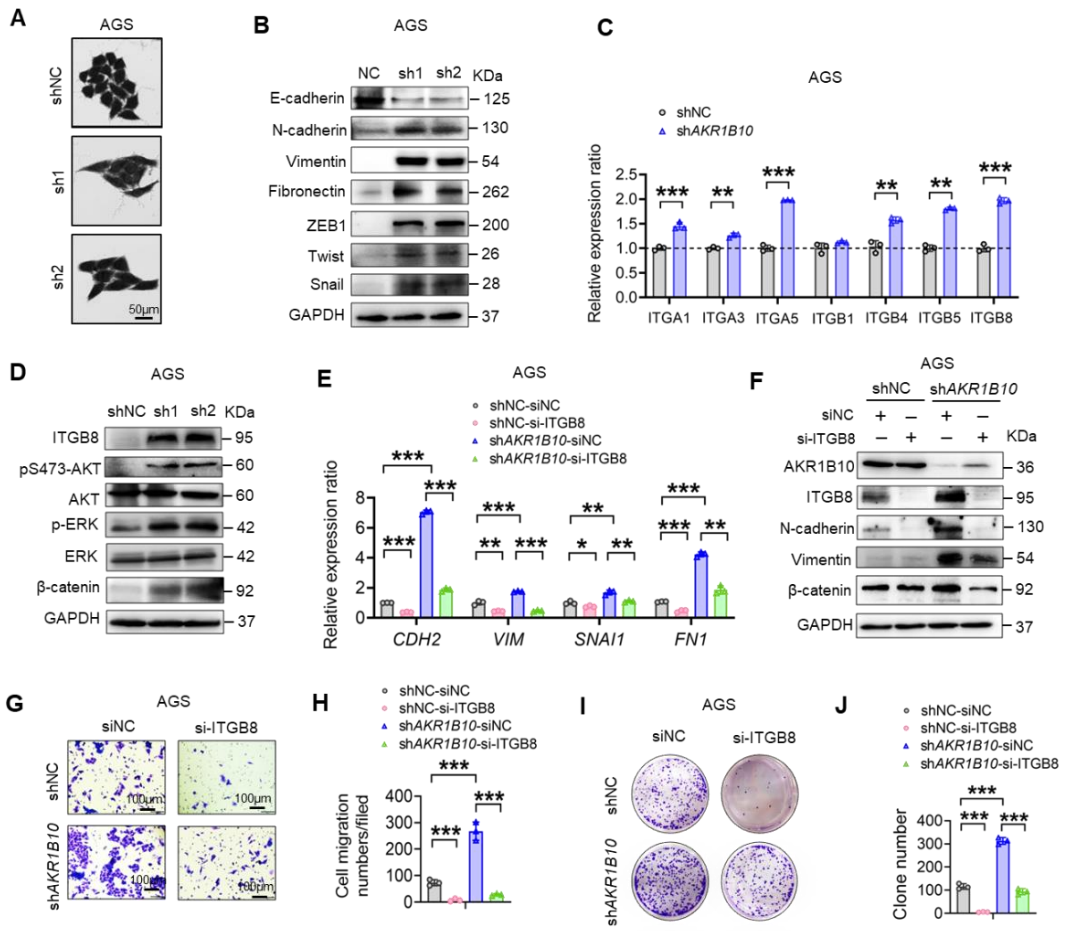

Fig. S4. AKR1B10 inhibits EMT by downregulating integrin signaling in GC cells. (**A**) Representative images for cell morphology of AGS (shNC&sh*AKR1B10*) cells. (**B**) Western blot analysis of EMT markers and EMT-related transcription factors expression in AGS cells. (**C**) qPCR analysis of integrin mRNA expression in AGS (shNC&sh*AKR1B10*) cells. (**D**)Western blot analysis of ITGB8 and its downstream factors expression in AGS cells. (**E**) qPCR analysis of the mRNA expression of EMT-related factors in AGS cells after ITGB8 silencing. (**F**) Western blot analysis of ITGB8 and its downstream EMT markers expression in AGS cells after ITGB8 silencing. (**G** and **H**) Representative images (G) and quantification (H) of transwell assays in AGS cells after ITGB8 silencing. (**I** and **J**) Representative images (I) and quantification (J) of clonogenic assays in AGS cells after ITGB8 silencing. Mean±SD, unpaired Student's *t* test [(C), (E), (H), and (J); n=3]. $*p < 0.05$, $**p < 0.01$, $***p < 0.001$

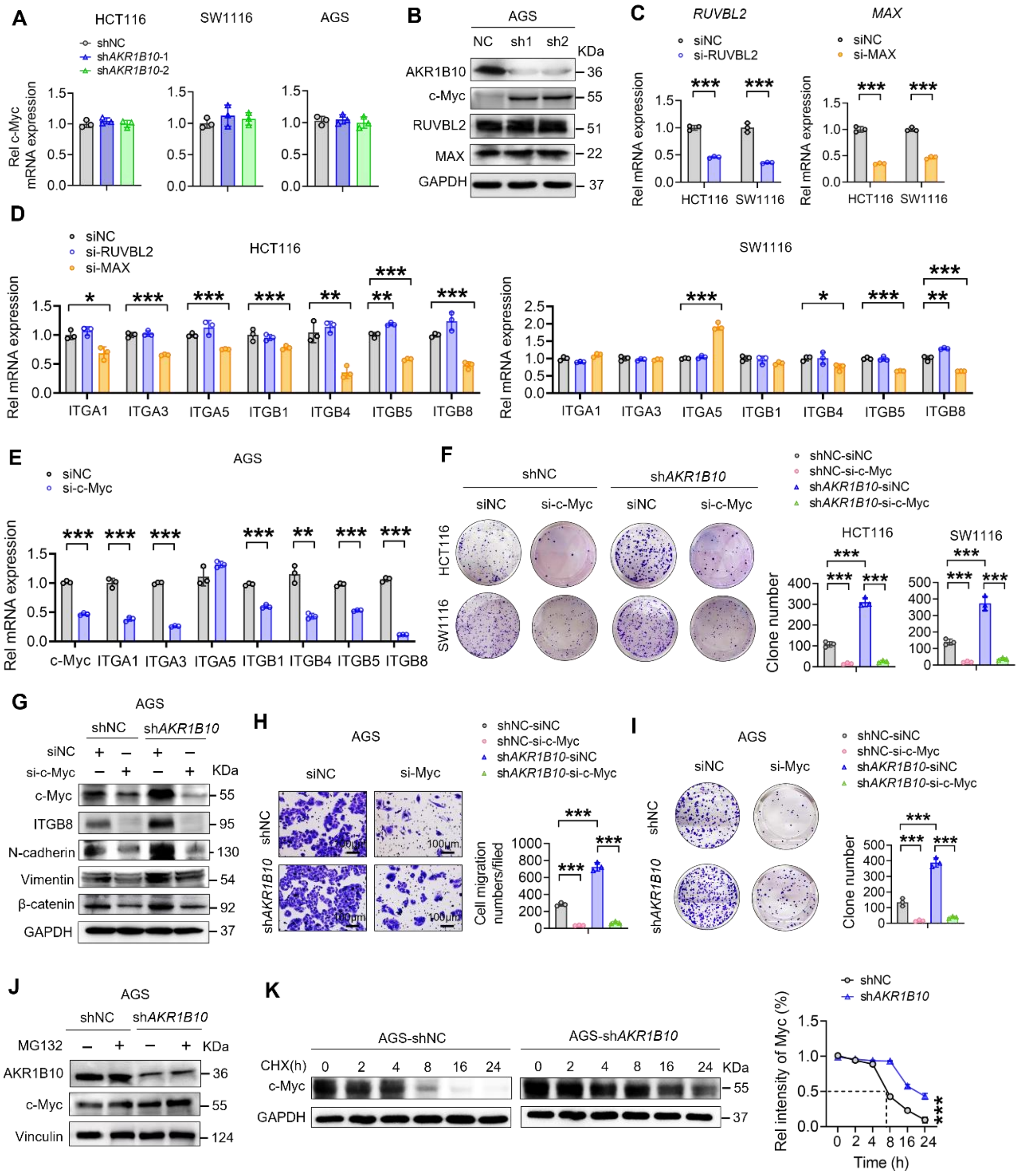

Fig. S5. AKR1B10 destabilizes c-Myc to suppress integrin transcription in CRC and GC cells. (**A**) qPCR analysis of c-Myc mRNA expression in HCT116, SW1116, and AGS (shNC&sh*AKR1B10*) cells. (**B**) Western blot analysis of c-Myc, RUVBL2, and MAX protein expression in AGS (shNC&sh*AKR1B10*) cells. (**C**) qPCR analysis of the knockdown efficiency of RUVBL2 and MAX in HCT116 and SW1116 cells after indicated siRNA transfection. (**D**) qPCR analysis of integrin mRNA expression in HCT116 and SW1116 cells after RUVBL2 and MAX silencing. (**E**) qPCR analysis of integrin mRNA expression in AGS cells after c-Myc silencing. (**F**) Representative images and quantification of clonogenic assays in HCT116 and SW1116 cells after c-Myc silencing. (**G**) Western blot analysis of ITGB8 and its downstream EMT markers expression in AGS cells after c-Myc silencing. (**H** and **I**) Representative images

and quantification of transwell assays (H) and clonogenic assays (I) in AGS cells after c-Myc silencing. (**J**) Western blot analysis of c-Myc expression in AGS (shNC&sh*AKR1B10*) cells treated with MG132 (10μM, 8h). (**K**) Detection of c-Myc protein turnover in AGS cells by western blot after CHX (100μg/ml) treatment and quantified by ImageJ software. Mean±SD, with unpaired Student's *t* test [(A), (C) to (F), (H) and (I); n=3], or two-way ANOVA (K, n=3). *$p < 0.05$, **$p < 0.01$, ***$p < 0.001$

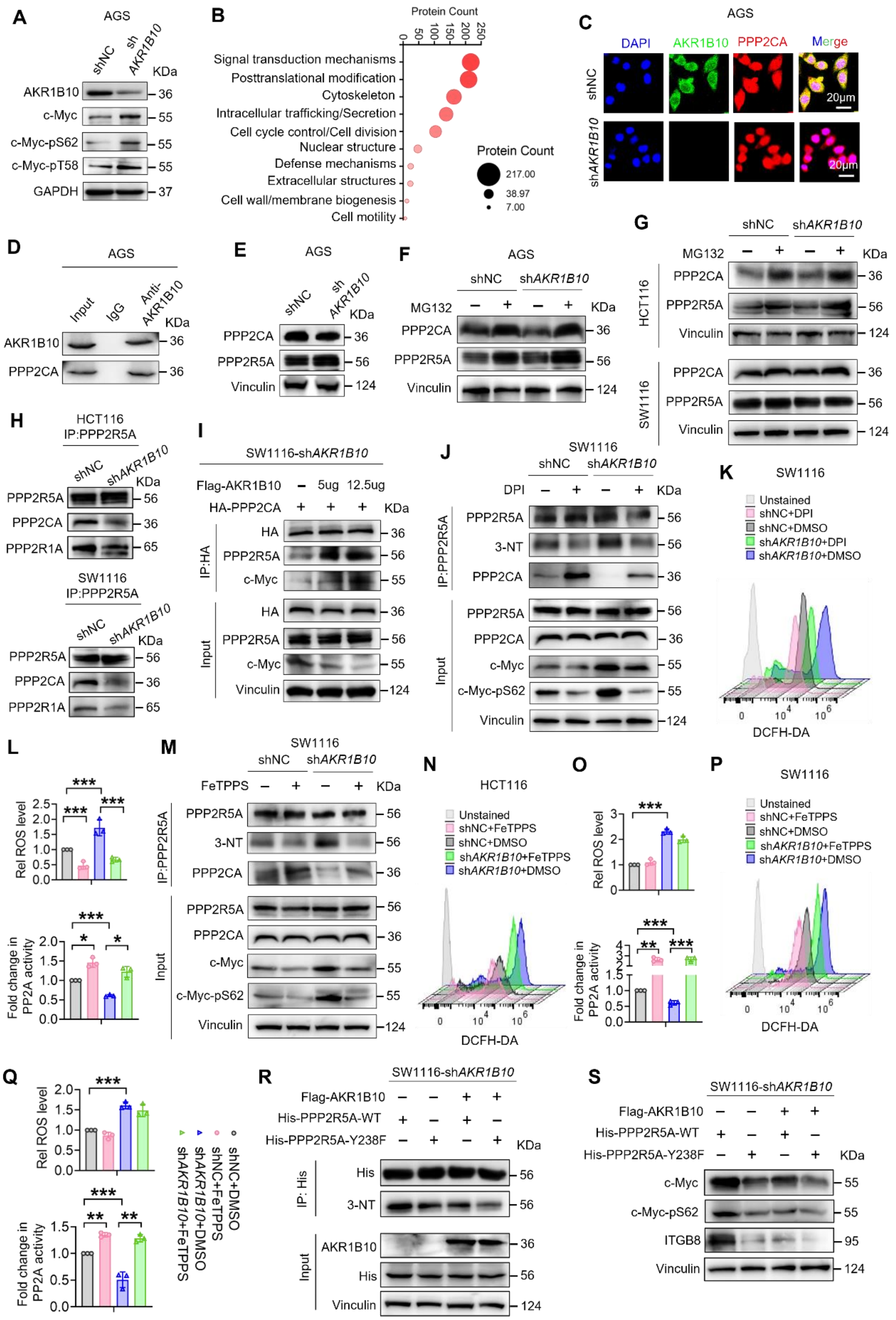
A
AGS
shNC
sh AKR1B10
KDa
AKR1B10
c-Myc
c-Myc-pS62
c-Myc-pT58
GAPDH
B
Protein Count
Signal transduction mechanisms
Posttranslational modification
Cytoskeleton
Intracellular trafficking/Secretion
Cell cycle control/Cell division
Nuclear structure
Defense mechanisms
Extracellular structures
Cell wall/membrane biogenesis
Cell motility
217.00
38.97
7.00
C
AGS
DAPI
AKR1B10
PPP2CA
Merge
shNC
shAKR1B10
20μm
D
AGS
Input
IgG
Anti-AKR1B10
PPP2CA
E
PPP2R5A
Vinculin
F
MG132
G
HCT116
SW1116
H
HCT116 IP:PPP2R5A
PPP2R1A
SW1116 IP:PPP2R5A
I
SW1116-shAKR1B10
Flag-AKR1B10
5ug
12.5ug
HA-PPP2CA
IP:HA
HA
Input
J
DPI
IP:PPP2R5A
3-NT
K
Unstained
shNC+DPI
shNC+DMSO
shAKR1B10+DPI
shAKR1B10+DMSO
DCFH-DA
L
Rel ROS level
Fold change in PP2A activity
M
FeTPPS
N
shNC+FeTPPS
shAKR1B10+FeTPPS
O
P
Q
R
His-PPP2R5A-WT
His-PPP2R5A-Y238F
IP: His
His
S
ITGB8

Fig. S6. AKR1B10 facilitates PP2A assembly to destabilize c-Myc in CRC and GC cells. (**A**) Western blot analysis of c-Myc expression, and its phosphorylation status in AGS (shNC&sh*AKR1B10*) cells. (**B**) KEGG pathway enrichment of the top 10 cellular pathways associated with AKR1B10 based on IP-MS analysis. (**C**) Immunofluorescence staining showing the co-localization of endogenous AKR1B10 with PPP2CA. Scale bars: 20μm. (**D**) Co-IP analysis showing the physical interaction of endogenous AKR1B10 with PPP2CA in AGS cells. (**E**) Western blot analysis of PPP2CA and PPP2R5A protein expression in AGS (shNC&sh*AKR1B10*) cells. (**F** and **G**) Western blot analysis of PPP2CA and PPP2R5A expression in AGS (F), HCT116 and SW1116 (G) (shNC&sh*AKR1B10*) cells treated with MG132 (10μM, 8h). (**H**) Western blot analysis of PPP2CA and PPP2R1A in anti-PPP2R5A immunoprecipitates from HCT116 and SW1116 cells. (**I**) Co-IP analysis of HA-PPP2CA in SW1116-sh*AKR1B10* cells co-expressing Flag-AKR1B10 with varying plasmid amounts, as indicated. (**J** and **M**) Co-IP analysis of PPP2R5A indicating reduced 3-NT levels and higher interaction with PPP2CA in the presence of DPI (15μM/20h, J) or FeTPPS (20μM/3h, M) in SW1116 (shNC&sh*AKR1B10)* cells. (**K** and **L**) Representative FACS images (K) and quantification of intracellular ROS levels, and relative PP2A activities (L) in SW1116 (shNC&sh*AKR1B10*) cells treated with DPI (15μM/20h). (**N** and **P**) Representative FACS images of intracellular ROS levels in HCT116 (N) and SW1116 (P) (shNC&sh*AKR1B10*) cells treated with FeTPPS (10 or 20μM/3h). (**O** and **Q**) Quantification of intracellular ROS levels and relative PP2A activities in HCT116 (O) and SW1116 (Q) (shNC&sh*AKR1B10*) cells treated with FeTPPS (10 or 20μM/3h). (**R** and **S**) His-PPP2R5A-WT or His-PPP2R5A-Y238F plasmid was transfected into SW1116-sh*AKR1B10* cells with or without Flag-AKR1B10. Co-IP analysis showing reduced 3-NT levels of His-PPP2R5A-Y238F (R). Western blot demonstrating decreased total c-Myc, pS62 c-Myc, and reduced ITGB8 expression in the presence of His-PPP2R5A-Y238F (S). Mean±SD, unpaired Student's *t* test [(L), (O), and (Q); n=3]. $^{*}p < 0.05$, $^{**}p < 0.01$, $^{***}p < 0.001$

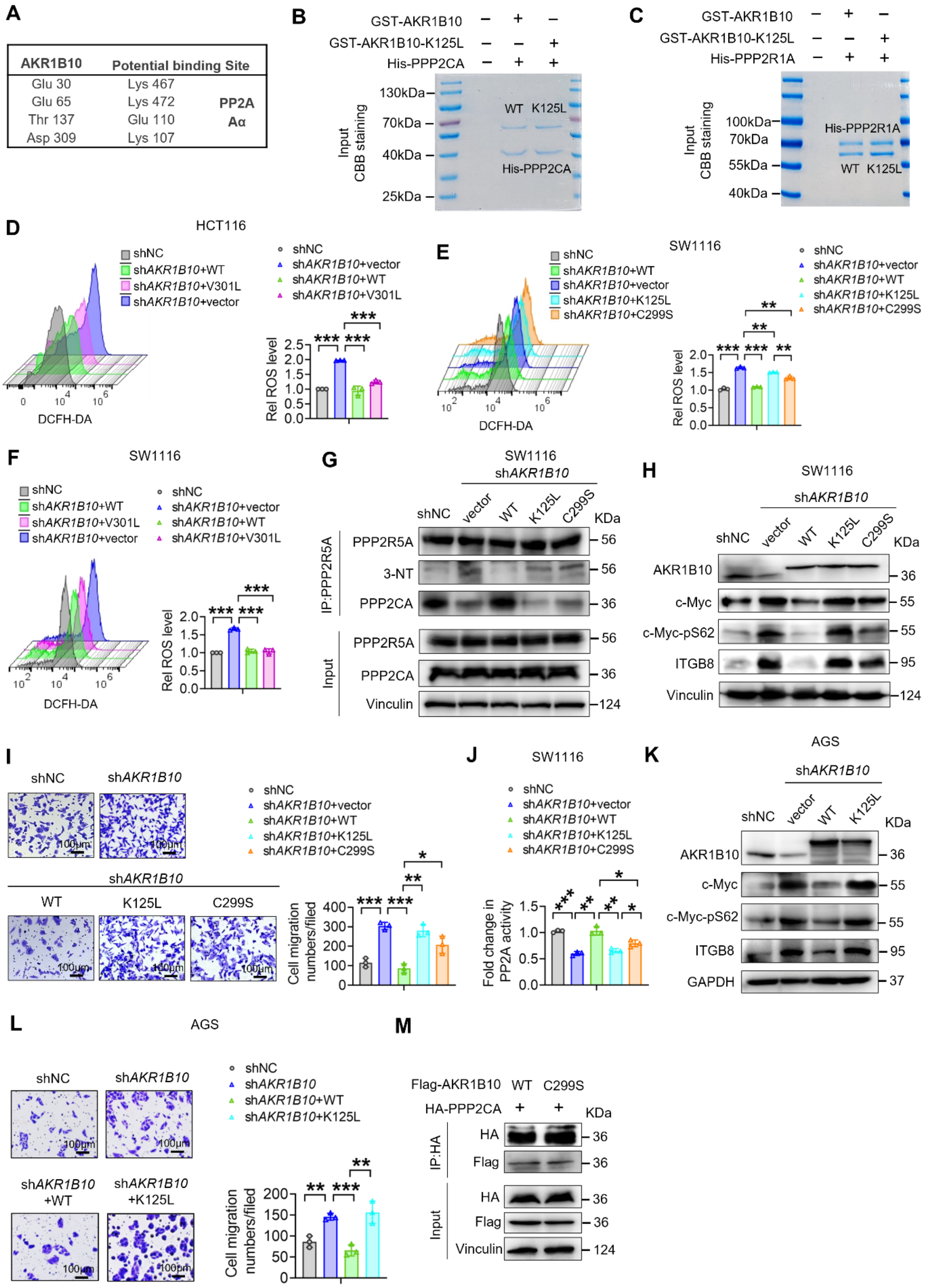
A
AKR1B10
Potential binding Site
Glu 30
Glu 65
Thr 137
Asp 309
Lys 467
Lys 472
Glu 110
Lys 107
PP2A Aα
B
GST-AKR1B10
GST-AKR1B10-K125L
His-PPP2CA
Input CBB staining
130kDa
70kDa
40kDa
25kDa
WT K125L
His-PPP2CA
C
GST-AKR1B10
GST-AKR1B10-K125L
His-PPP2R1A
Input CBB staining
100kDa
70kDa
55kDa
40kDa
His-PPP2R1A
WT K125L
D
HCT116
shNC
shAKR1B10+WT
shAKR1B10+V301L
shAKR1B10+vector
DCFH-DA
Rel ROS level
E
SW1116
shNC
shAKR1B10+WT
shAKR1B10+vector
shAKR1B10+K125L
shAKR1B10+C299S
DCFH-DA
Rel ROS level
F
SW1116
shNC
shAKR1B10+WT
shAKR1B10+V301L
shAKR1B10+vector
DCFH-DA
Rel ROS level
G
SW1116
shAKR1B10
shNC vector WT K125L C299S KDa
IP:PPP2R5A
PPP2R5A
3-NT
PPP2CA
Input
PPP2R5A
PPP2CA
Vinculin
H
SW1116
shAKR1B10
shNC vector WT K125L C299S KDa
AKR1B10
c-Myc
c-Myc-pS62
ITGB8
Vinculin
I
shNC
shAKR1B10
WT
K125L
C299S
100μm
shNC
shAKR1B10+vector
shAKR1B10+WT
shAKR1B10+K125L
shAKR1B10+C299S
Cell migration numbers/filed
J
SW1116
shNC
shAKR1B10+vector
shAKR1B10+WT
shAKR1B10+K125L
shAKR1B10+C299S
Fold change in PP2A activity
K
AGS
shAKR1B10
shNC vector WT K125L KDa
AKR1B10
c-Myc
c-Myc-pS62
ITGB8
GAPDH
L
AGS
shNC
shAKR1B10
shAKR1B10 +WT
shAKR1B10 +K125L
100μm
Cell migration numbers/filed
M
Flag-AKR1B10 WT C299S
HA-PPP2CA + +
KDa
IP:HA
HA
Flag
Input
HA
Flag
Vinculin

Fig. S7. AKR1B10^{K125L} promotes CRC metastasis by disrupting PP2A assembly. (**A**) Computational docking model predicting the potential binding sites between AKR1B10 and PPP2R1A. (**B** and **C**) Coomassie brilliant blue (CBB) staining of the input proteins for GST pulldown assays with recombinant GST-tagged AKR1B10 (WT or K125L mutant) and His-PPP2CA (B) or His-PPP2R1A (C). (**D**) Representative FACS images (left) and quantification of intracellular ROS levels (right) in HCT116 shNC and sh*AKR1B10* cells expressing mock, AKR1B10^{WT}, AKR1B10^{V301L}. (**E** and **F**) Representative FACS images (left) and quantification of intracellular ROS levels (right) in SW1116 shNC and sh*AKR1B10* cells expressing mock, AKR1B10^{WT}, AKR1B10^{K125L}, AKR1B10^{C299S} (E) and AKR1B10^{V301L} (F). (**G**) Immunoprecipitation of PPP2R5A demonstrating increased 3-NT and reduced PPP2CA interaction in AKR1B10^{K125L} and AKR1B10^{C299S} overexpressing SW1116-sh*AKR1B10* cells. (**H**) Western blot analysis of c-Myc, pS62 c-Myc, and ITGB8 expression in SW1116 shNC and sh*AKR1B10* cells expressing mock, AKR1B10^{WT}, AKR1B10^{K125L}, AKR1B10^{C299S}. (**I** and **J**) Representative images (left) and quantification (right) of transwell assays (I), and relative PP2A activities (J) in SW1116 shNC and sh*AKR1B10* cells expressing mock, AKR1B10^{WT}, AKR1B10^{K125L}, AKR1B10^{C299S}. (**K**) Western blot analysis of c-Myc, pS62 c-Myc, and ITGB8 expression in AGS shNC and sh*AKR1B10* cells expressing mock, AKR1B10^{WT}, and AKR1B10^{K125L}. (**L**) Representative images (left) and quantification (right) of transwell assays in AGS shNC and sh*AKR1B10* cells expressing mock, AKR1B10^{WT}, and AKR1B10^{K125L}. (**M**) Co-IP analysis showing the interaction between PPP2CA with AKR1B10^{C299S} in HCT116 cells, using an anti-HA antibody. Mean±SD, unpaired Student's *t* test [(D) to (F), (I), (J), and (L); n=3]. *$p < 0.05$, **$p < 0.01$, ***$p < 0.001$

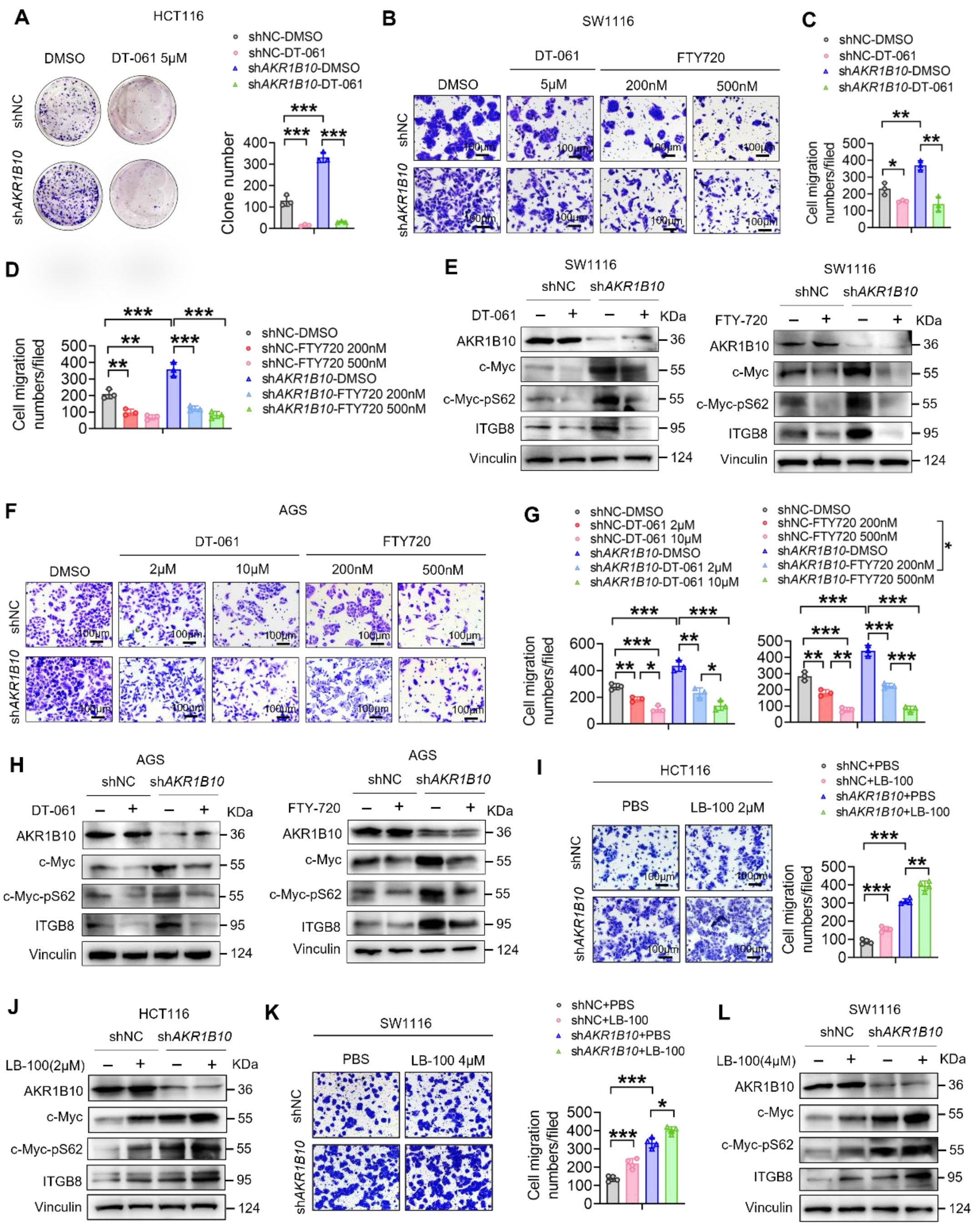

Fig. S8. Pharmacological restoring PP2A activity suppressed AKR1B10 loss-driven metastasis. (**A**) Representative images and quantification of clonogenic assays in HCT116 cells treated with DT-061. (**B-D**) Representative images (B) and quantification (C, D) of transwell assays in SW1116 cells treated with DT-061 or FTY-720. (**E**) Western blot analysis of c-Myc, pS62 c-Myc, and ITGB8 protein expression in SW1116 cells treated with DT-061 or FTY-720. (**F** and **G**) Representative images (F) and quantification (G) of transwell assays in AGS cells treated with DT-061 or FTY-720. (**H**) Western blot analysis of c-Myc, pS62 c-Myc, and ITGB8 expression in AGS cells treated with DT-061 or FTY-720. (**I** and **K**) Representative images and quantification of transwell assays in HCT116 (I; 2 μM LB-100, 48h) and SW1116 (K; 4 μM LB-

100, 48h) cells. (**J** and **L**) Western blot analysis of c-Myc, pS62 c-Myc, and ITGB8 expression in HCT116 (J) and SW1116 (L) cells treated with LB-100 for 24h. [(A), (C), (D), and (G)] n = 3; [(I) and (K)] n=4. Mean±SD, unpaired Student's *t* test [(A), (C), (D), (G), (I) and (K)]. *$p < 0.05$, **$p < 0.01$, ***$p < 0.001$

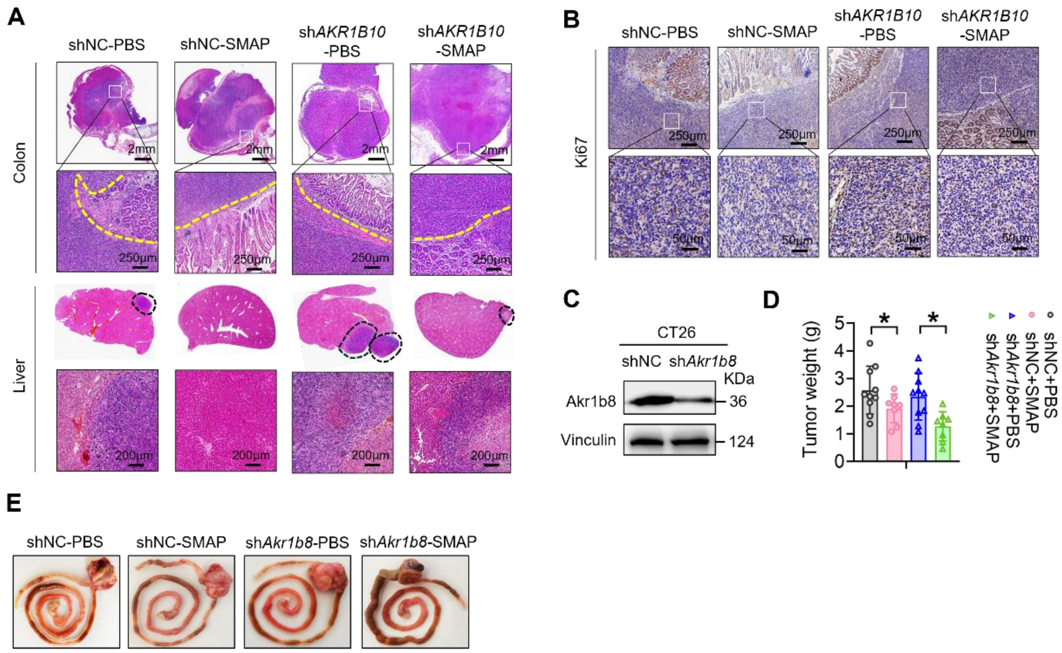

Fig. S9. Pharmacological restoring PP2A activity suppressed AKR1B10 loss-driven metastasis *in vivo*. (**A** and **B**) HCT116 orthotopic implantation mouse models treated with SMAP via gavage at 5mg/kg on alternate days (n=8 mice per group). Representative H&E staining of primary tumors and liver metastases (A). Representative IHC images of Ki67 in primary tumors (B, n=5 mice). (**C**) Western blot analysis of *Akr1b8* knockdown efficiency in CT26 cells. (**D** and **E**) CT26 orthotopic implantation models treated with SMAP via gavage at 5mg/kg on alternate days (n=8-10 mice per group). Quantification of primary tumor weights (D) and representative images of primary tumors (E). Mean±SD, unpaired Student's *t* test (D). *$p < 0.05$

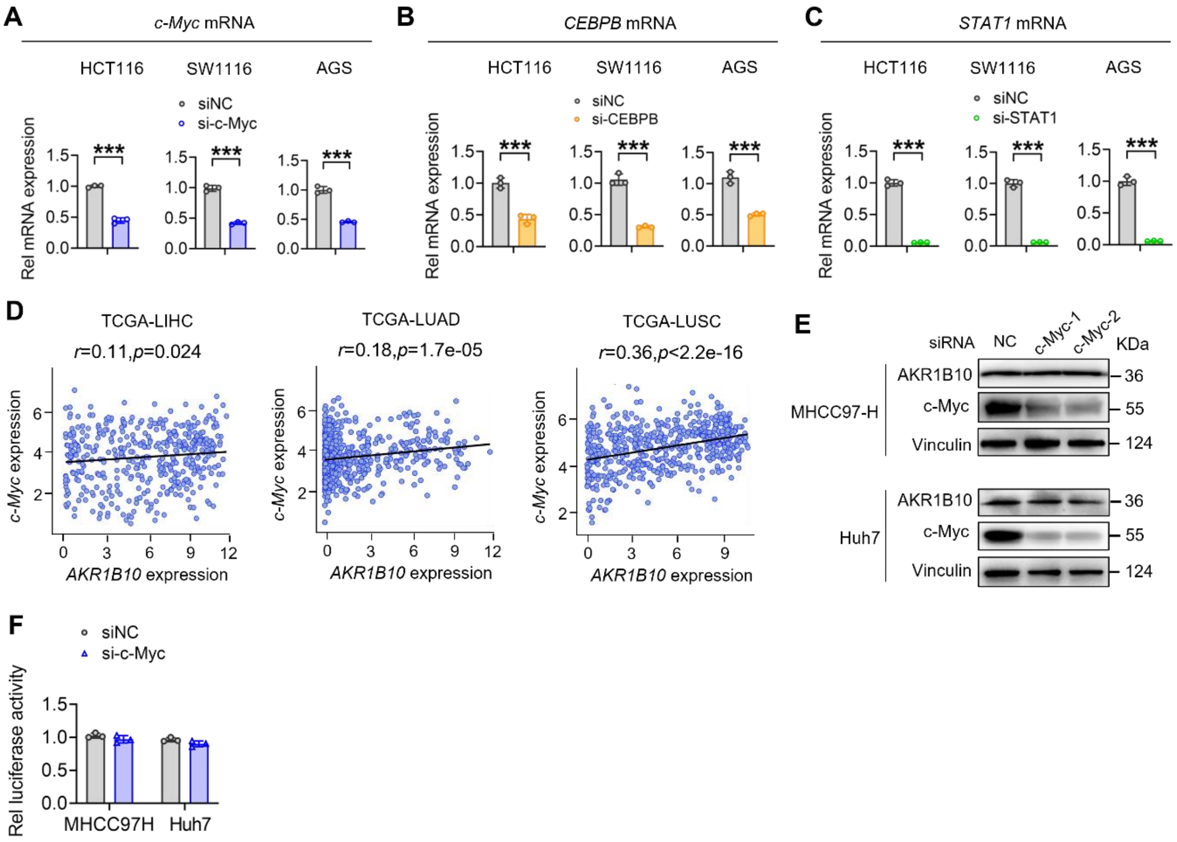

Fig. S10. Knockdown c-Myc enhanced *AKR1B10* transcription in CRC/GC cells, but not HCC. (**A**) qPCR analysis of c-Myc mRNA expression in HCT116, SW1116, and AGS cells after c-Myc silencing. (**B**) qPCR analysis of CEBPB mRNA expression in HCT116, SW1116, and AGS cells after CEBPB silencing. (**C**) qPCR analysis of STAT1 mRNA expression in HCT116, SW1116, and AGS cells after STAT1 silencing. (**D**) Correlation between AKR1B10 and c-Myc mRNA levels in human LIHC (n=419), LUAD (n=571), and LUSC (n=547) tissues, based on TCGA database. (**E**) Western blot analysis of AKR1B10 protein expression in MHCC97-H and Huh7 cells after c-Myc knockdown. (**F**) Dual-luciferase reporter assay of *AKR1B10* promoter activity in MHCC97-H and Huh7 cells after c-Myc knockdown. Mean±SD, unpaired Student's *t* test [(A) to (C); n=3]. ***$p < 0.001$

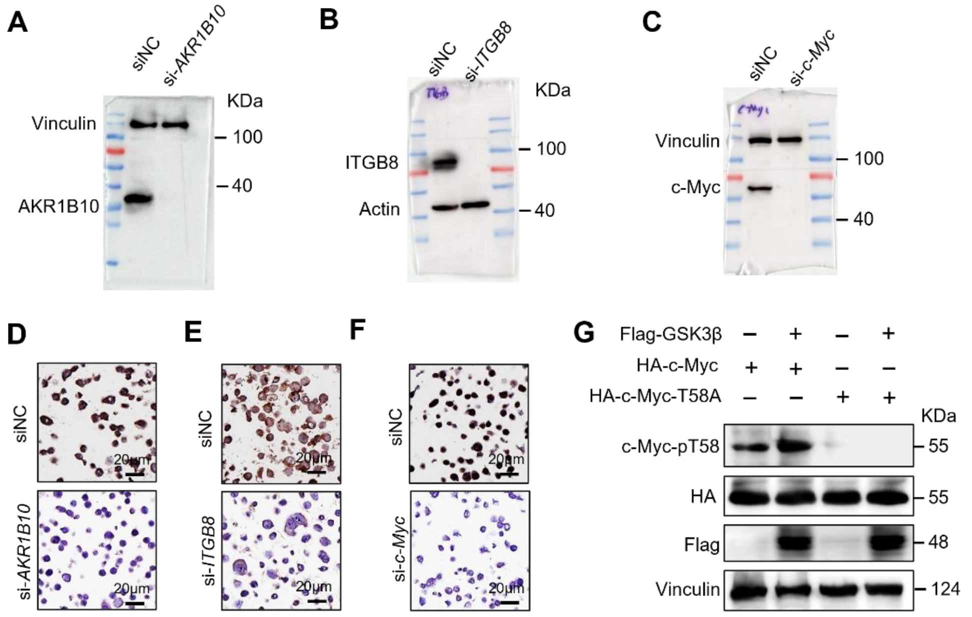

Fig. S11. Antibody specificity validation. (**A-C**) Western blot analysis of AKR1B10 (A), ITGB8 (B), and c-Myc (C) expression in HCT116 cells following siRNA-mediated knockdown (representative full-length gels shown). (**D-F**) Corresponding IHC staining of paraffin-embedded HCT116 cell pellets using anti-AKR1B10 (Signalway Antibody, 36071, D), anti-ITGB8 (Proteintech, 29775-1-AP, E), and anti-c-Myc (Abcam, ab32072, F). (**G**) HCT116 cells were co-transfected with Flag-GSK3β and either HA-c-Myc or HA-c-Myc-T58A and then subjected to western blot using pT58-MYC antibody (AP0990, ABclonal).

Table S1. Clinical features of CRC patients in TMAs

Clinical feature	Group	Low expression (n=130)	%	High expression (n=87)	%
Age	≤55	47	36.2	26	29.9
	>55	83	63.8	61	70.1
Gender	Men	78	60.0	50	57.5
	Women	52	40.0	37	42.5
Localization	Colon	82	63.1	57	65.5
	Rectum	48	36.9	30	34.5
Differentiation	Moderately	42	32.3	25	28.7
	Poorly	11	8.5	8	9.2
	Missing	77	59.2	54	62.1

Table S2. Site-directed mutagenesis primers for AKR1B10/PPP2R5A

Primer name	Sequence
hAKR1B10-EcoRI Fw	cctcaGAATTCGCCACCATGGCCACGTTTGTG
hAKR1B10-SacII Rw	cctcaCCGCGGGAAGGAAGGTCCGCTGGATT
hAKR1B10-K125L Rw	GGCATTACCTTTATCATCTAGGGGGAAAAGGTCATC
hAKR1B10-K125L Fw	GATGACCTTTTCCCCCTAGATGATAAAGGTAATGCC
hAKR1B10-P219S Rw	TGGGCTCTCCGGATAGATCTTGGGCCAAGCCAGAAGAC
hAKR1B10-P219S Fw	GTCTTCTGGCTTGGCCCAAGATCTATCCGGAGAGCCCA
hAKR1B10-K263R Rw	GCGTGCTGGTGTCACAGACCTGGGGATGACAATCACAT
hAKR1B10-K263R Fw	ATGTGATTGTCATCCCCAGGTCTGTGACACCAGCACGC
hAKR1B10-C299S Fw	AGAGGATTGCAACACGTTACTGGCCCTCCAGTTTCTGT
hAKR1B10-C299S Rw	ACAGAAACTGGAGGGCCAGTAACGTGTTGCAATCCTCT
hAKR1B10-V301L Rw	CTGGAGGGCCTGTAACCTGTTGCAATCCTCTCATTTG
hAKR1B10-V301L Fw	CAAATGAGAGGATTGCAACAGGTTACAGGCCCTCCAG
hPPP2R5A-BamHI Fw	cctcaGGATCCATGTCGTCGTCGTCGCCGC
hPPP2R5A-Xhol Rw	cctcaCTCGAGTTCGGCACTTGTATTGCTGAGAATAC
hPPP2R5A-Y238F Fw	CATTTTCCTCAGGTTTATATTTGAAACAGAACATTTCAAT GGTG
hPPP2R5A-Y238F Rw	CACCATTGAAATGTTCTGTTTCAAATATAAACCTGAGGAA AATG

Table S3. The sequences of shRNA and siRNA

Gene name	Targeted sequences	Region
AKR1B10	GCCTATGTCTATCAGAATGAA	CDS
AKR1B10	GCACGCATTGTTGAGAACATT	CDS
Akr1b8	GCCATCACGTATACAGGAGAA	CDS
Akr1b8	CCTCACCAGTAAGACAACATT	CDS
ITGB8	CAGCACTGTGTCAATTCAA	CDS
MYC	CCTGAGACAGATCAGCAACAA	CDS
MYC	CAGTTGAAACACAAACTTGAA	CDS
MAX	ACACACACCAGCAAGATATTG	CDS
RUVBL2	CGAGAAAGACACGAAGCAGAT	CDS
CEBPB	CCCGTGGTGTTATTTAAAGAA	CDS
STAT1	CTGGAAGATTTACAAGATGAA	CDS

Table S4. Primer sequences for qRT-PCR

Gene name	Primer	Sequence
AKR1B10	Fw	AACGTGTTGCAATCCTCTCA
	Rw	TGGGACATGAGTGGAGGTAGT
Akr1b8	Fw	TGAGGATTCTGCCTTGGTCAT
	Rw	ACGGATCTGAAGCTGGACTATTT
ITGA1	Fw	GGACAGCCTCGGTACAATCA
	Rw	CTGCCAAAGTAGGAACCAATCT
ITGA3	Fw	CAGTGAGTCCGCTGTCTTCCA
	Rw	TCCGCAAAGGTAAAGAGTAGTTCA
ITGA5	Fw	GGGTGGCCTTCGGTTTACAG
	Rw	CGCTTTGCGAGTTGTTGAGATT
ITGB1	Fw	ATGCCTACTTCTGCACGATG
	Rw	TCCTTTGCTACGGTTGGTTA
ITGB4	Fw	TGCTTATTGAGAACCTTCGGGAGT
	Rw	CACGATAGGGATGTCAGGGATG
ITGB5	Fw	CTGGAACAACGGTGGAGATT
	Rw	TGGCAGGTAGCAGTAAAGAAGA
ITGB8	Fw	GTTACATTCTTGATTGGGTTGC
	Rw	CGTCGGTAGGTGACTGCTCT
CDH2	Fw	AAAGAACGCCAGGCCAAACAAC
	Rw	CCATTCGTCGGATTCCCACAGG
VIM	Fw	ATCTGGATTCACTCCCTCTGGTTG
	Rw	CATCGTGATGCTGAGAAGTTTCG
SNAI1	Fw	CCCTCAAGATGCACATCCGAAGC
	Rw	TTGGAGCGGTCAGCGAAGGCAC
FN1	Fw	GCCAACCTTTACAGACCTATCC
	Rw	CTTCCAACGGCCTACAGAATT
MYC	Fw	GGAGGAACAAGAAGATGAGGAAG
	Rw	AGGACCAGTGGGCTGTGAGG
GAPDH	Fw	GGACTCATGACCACAGTCCA
	Rw	AGGCAGGGATGATGTTCTGG

Table S5. Primer sequences for ChIP-qPCR

Gene name	Primer	Sequence
AKR1B10-ChIP	Fw	CAACCAAAGCCAACTCATCAAC
(-850~-861)	Rw	GGTAGTCAGCAATCTAGGGAACA
AKR1B10-ChIP	Fw	TTCCTCATAATCTGGTGGCC
(-1422~-1433)	Rw	AATACTAGCACTGCATGTCA
AKR1B10-ChIP	Fw	CCTTCCCTGACATCCCAGTC
(-2181~-2192)	Rw	CCATTTATTTGCTTGTGATTTTG

Table S6. Antibody list

Antibody name	Company	Host Species	Cat No.	Usage
AKR1B10	ABclonal	Rabbit	A7823	WB, IP
AKR1B10	Signalway	Rabbit	36071	IHC, IF
E-cadherin	Cell signaling	Rabbit	3195	WB
N-cadherin	Cell signaling	Rabbit	4061	WB
Vimentin	Santa cruz	Mouse	sc-6260	WB
Fibronectin	ABclonal	Rabbit	A12932	WB
ZEB1	Cell signaling	Rabbit	3396	WB
Twist	Santa cruz	Mouse	sc-81417	WB
Snail	Proteintech	Rabbit	29775-1-AP	WB
ITGB8	Santa Cruz	Mouse	sc-7966	WB
ITGB8	Proteintech	Rabbit	29775-1-AP	IHC
pAKT (Ser473)	Cell signaling	Rabbit	4060	WB
AKT	ABclonal	Rabbit	A18675	WB
pErk1/2 (Thr202/Tyr204)	Cell signaling	Rabbit	4370	WB
Erk1/2	Cell signaling	Rabbit	4675	WB
β-Catenin	Cell signaling	Rabbit	9562	WB
c-MYC	Proteintech	Mouse	67447-1-Ig	WB
c-MYC	Abcam	Rabbit	ab32072	WB, IHC
RUVBL2	ABclonal	Rabbit	A12564	WB
MAX	Proteintech	Rabbit	10426-1-AP	WB
p-c-Myc (Thr58)	ABclonal	Rabbit	AP0990	WB
p-c-Myc (Ser62)	Cell signaling	Rabbit	13748	WB
PPP2CA	ABclonal	Rabbit	A6702	WB, IF
PPP2R5A	Proteintech	Rabbit	12675-2-AP	WB, IP
3-Nitrotyrosine	MCE	Rabbit	HY-P81216	WB
Acetylated-Lysine	Cell signaling	Rabbit	9441	WB
Phospho-(Ser/Thr)	Abcam	Rabbit	ab17464	WB
Ki67	Abcam	Rabbit	ab15580	IHC
HA tag	Proteintech	Rabbit	51064-2-AP	WB, IP
Flag tag	Sigma Aldrich	Mouse	F1804	WB, IP
Vinculin	ABclonal	Rabbit	A2752	WB
GAPDH	ABclonal	Mouse	AC054	WB
β-Actin	Cell signaling	Rabbit	4967	WB
HRP-mouse IgG	Cell signaling	Goat	7076	WB
HRP-rabbit IgG	Cell signaling	Goat	7074	WB
